# Identification and validation of a copper homeostasis-related gene signature for the predicting prognosis of breast cancer patients via integrated bioinformatics analysis

**DOI:** 10.1038/s41598-024-53560-9

**Published:** 2024-02-07

**Authors:** Yi Li, Xiuxian Wei, Yuning Wang, Wenzhuo Wang, Cuntai Zhang, Deguang Kong, Yu Liu

**Affiliations:** 1 Department of Geriatrics, Tongji Hospital of Tongji Medical College, Huazhong University of Science and Technology, Building 6, 1095 Jiefang Avenue, Wuhan, 430030 People’s Republic of China; 2 Key Laboratory of Vascular Aging, Ministry of Education, Tongji Hospital of Tongji Medical College, Huazhong University of Science and Technology, Wuhan, 430030 People’s Republic of China; 3 Department of Breast and Thyroid Surgery, Renmin Hospital of Wuhan University, 238 Ziyang Road, Wuhan, 430060 People’s Republic of China

**Keywords:** Copper homeostasis, lncRNAs, Breast cancer, Immune infiltration, Tumor microenvironment, Cancer genetics, Cancer microenvironment, Tumour biomarkers, Cancer, Genetics

## Abstract

The prognostic value of copper homeostasis-related genes in breast cancer (BC) remains largely unexplored. We analyzed copper homeostasis-related gene profiles within The Cancer Genome Atlas Program breast cancer cohorts and performed correlation analysis to explore the relationship between copper homeostasis-related mRNAs (chrmRNA) and lncRNAs. Based on these results, we developed a gene signature-based risk assessment model to predict BC patient outcomes using Cox regression analysis and a nomogram, which was further validated in a cohort of 72 BC patients. Using the gene set enrichment analysis, we identified 139 chrmRNAs and 16 core mRNAs via the Protein–Protein Interaction network. Additionally, our copper homeostasis-related lncRNAs (chrlncRNAs) (PINK1.AS, OIP5.AS1, HID.AS1, and MAPT.AS1) were evaluated as gene signatures of the predictive model. Kaplan–Meier survival analysis revealed that patients with a high-risk gene signature had significantly poorer clinical outcomes. Receiver operating characteristic curves showed that the prognostic value of the chrlncRNAs model reached 0.795 after ten years. Principal component analysis demonstrated the capability of the model to distinguish between low- and high-risk BC patients based on the gene signature. Using the pRRophetic package, we screened out 24 anticancer drugs that exhibited a significant relationship with the predictive model. Notably, we observed higher expression levels of the four chrlncRNAs in tumor tissues than in the adjacent normal tissues. The correlation between our model and the clinical characteristics of patients with BC highlights the potential of chrlncRNAs for predicting tumor progression. This novel gene signature not only predicts the prognosis of patients with BC but also suggests that targeting copper homeostasis may be a viable treatment strategy.

## Introduction

Breast cancer (BC) is the most prevalent malignancy among women in the United States, with over 4 million patients diagnosed with invasive breast cancer^1^. Key molecular targets, such as estrogen receptor-alpha (ERα), progesterone receptor (PR), and epidermal growth factor-2 (ERBB2), have been identified and guide therapeutic strategies^2^. For non-metastatic patients, a combination of surgical resection and systemic therapies, including neoadjuvant and adjuvant chemotherapy, targeted therapy, endocrine therapy, and adjuvant radiation, is recommended^2^. However, the prognosis of advanced breast cancer remains poor, with the 5-year survival rate decreasing to 28% after local invasion and distant metastasis^1^. Despite rapid advancements in targeted therapies that benefit some patients, universally applicable prognostic biomarkers are critically needed to stratify risks and guide treatment decisions. Mutations in BRCA1 and BRCA2 are the most significant DNA alterations associated with effective targeted therapies for breast cancer^3^. Additionally, biomarker panels combining specific gene groups have proven more diagnostically powerful than individual markers identified in traditional clinical trials^4^. Therefore, identifying precise signatures that can accurately predict breast cancer prognosis is urgently needed to enhance patient outcomes.

Programmed cell death (PCD) is universally acknowledged as a key player in morphogenesis and homeostasis maintenance^5^. Recently, attention has shifted towards a novel cell death pathway induced by intracellular copper (Cu), known as cuprotosis^6^. Copper is a vital micronutrient that is critical in various biological processes, including iron transport, oxygen transport, and energy metabolism. Disruption of copper homeostasis can lead to the accumulation of lipoylated mitochondrial components, triggering proteotoxic stress and subsequent cell death. Considering the crucial role of glycolysis in cancer cell survival and proliferation, copper-based therapies may reduce the malignant potential of cancer cells. Emerging evidence emphasizes the growing importance of coproptosis-related genes in the clinical diagnosis and treatment of breast cancer^7^. The copper ionophore elelesclomol, which transports copper ions, disrupts the FDX1-mediated synthesis of Fe–S cluster proteins. This disruption leads to their accumulation in the mitochondria and induces cytoproptosis in tumor cells^8^. The focused identification and targeting of these critical genes and the mechanisms related to cuproptosis are becoming central to breast cancer research. Delving deeper into the mechanisms of cuproptosis in breast cancer and developing novel drugs targeting this pathway are urgently needed to enhance the prognosis of patients with breast cancer.

Long noncoding RNAs (lncRNAs), which are functional RNA molecules exceeding 200 nucleotides in length, play critical roles in gene transcription and post-transcriptional modification despite their lack of protein-coding potential. Recently, lncRNAs have been identified as promising biomarkers for diagnosing and prognosing various diseases^9^. Aberrant expression of specific lncRNAs promotes tumor development and is linked to poor clinical outcomes. Several studies have suggested that Cu-related lncRNAs have prognostic value in renal cancer^10,11^, liver cancer^12^, and melanoma^13^. However, the implications of these Cu-regulating lncRNAs in breast cancer are not fully understood. Advances in high-throughput research methods have greatly facilitated the systematic identification and characterization of these lncRNAs, laying the groundwork for further investigations. Notably, researchers have increasingly utilized a combination of bioinformatics and machine learning algorithms. These methods allow for a more holistic understanding of the intricate molecular networks involved in cancer progression and raise the potential for establishing lncRNA prognostic signatures that could guide targeted therapies.

In our study, we explored the (TCGA) BRCA cohort and identified 139 copper homeostasis-related genes, subsequently narrowing down to 13 key genes through Protein–Protein Interaction Network (PPI) network analysis. We identified four lncRNAs associated with these genes, referred to as copper homeostasis-related lncRNAs (chrlncRNAs), and constructed a prognostic model. We assessed the predictive value of chrlncRNAs in BC patients using functional enrichment and Cox regression analyses. We carried out a drug sensitivity analysis to determine the effectiveness of specific anticancer drugs in both high- and low-risk populations. This approach aids in bridging the gap between basic research and clinical applications by identifying how different risk groups respond to various treatments. We validated the chrlncRNA gene signature model using data from 72 patients with primary breast cancer. In tumor tissues, the four chrlncRNAs were highly expressed, and advanced clinical characteristics were closely associated with high-risk scores based on the gene signature. Our findings underscore the crucial role of copper homeostasis-related genes in BC development and their potential as therapeutic targets.

## Results

### Identification of the copper homeostasis and ATP transportation-related DEGs from TCGA BRCA cohort

**Figure 1:**
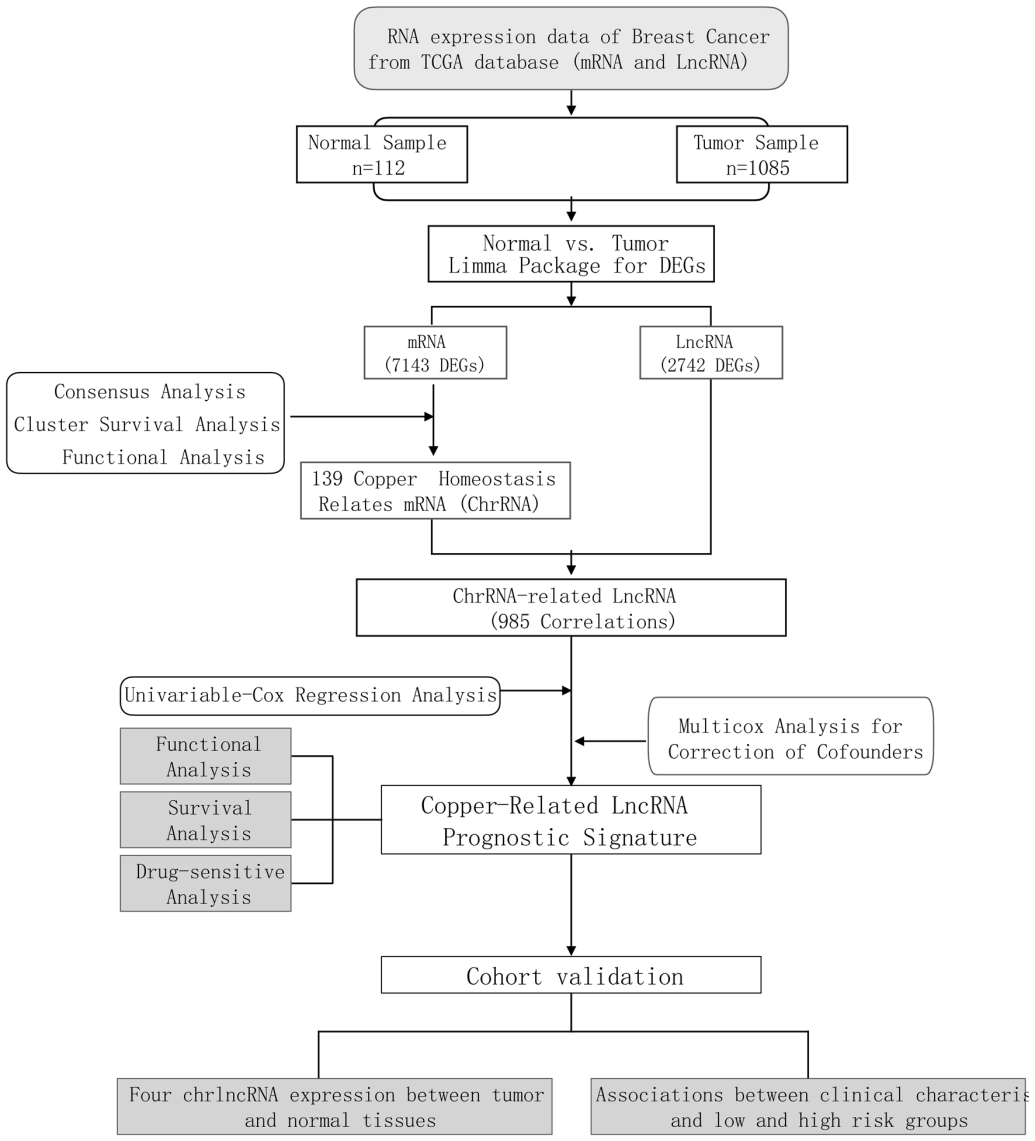
Flow chart showing the process of identifying copper homeostasis-related lncRNAs.

**Figure 2:**
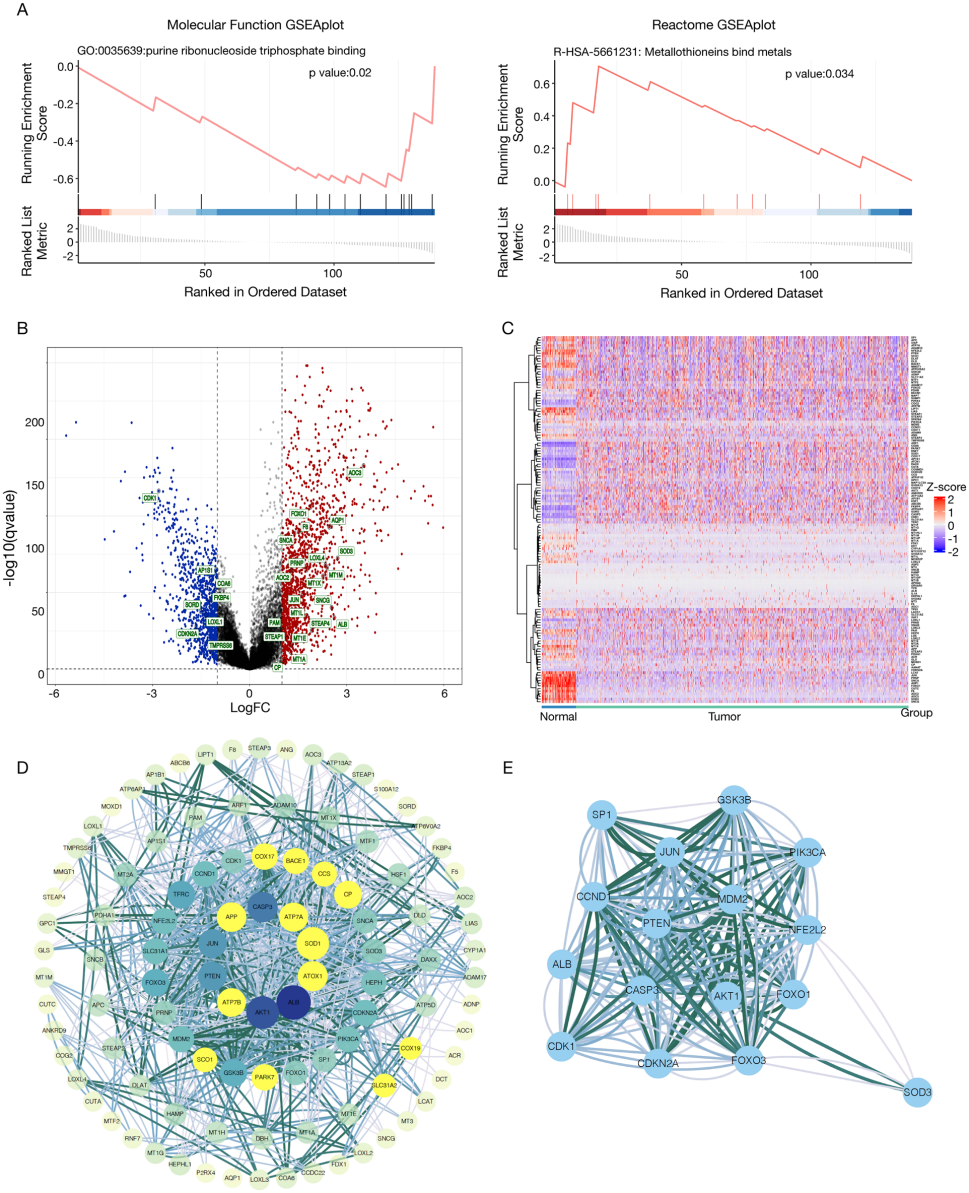
Expression of DEGs in the TCGA BRCA cohort and interactions among copper homeostasis-related genes. (**A**) Enrichment plots for the purine ribonucleoside triphosphate binding pathway and metallothioneins bind metals pathway; (**B**) Volcano plots of DEGs in the TCGA BRCA cohort, in which copper homeostasis-related mRNAs (chrmRNAs) were tagged by the standard of the absolute value of LogFC > =1 and FDR < 0.05. Red and blue dots indicate upregulated and downregulated genes, respectively; (**C**) A heatmap illustrating the distribution of chrmRNAs in normal and tumor tissue samples; (**D**) PPI network depicting chrmRNAs-related DEGs interactions, visualized using Cytoscape with genes arranged by correlation degree. Each concentric ring represents a 10-unit increase in correlation degree; (**E**) Identification of 16 hub genes using MCODE, visualized using Cytoscape.

A study flowchart is shown in Fig. 1. To explore differentially expressed genes (DEGs) between normal and breast cancer tissues, 112 healthy subjects and 1085 breast cancer patients were recruited from the TCGA BRCA cohort. A total of 7143 DEGs at the mRNA level and 2742 DEGs at the lncRNA level were identified, adhering to the criteria of a false discovery rate (FDR) < 0.05 and an absolute log fold change (|logFC|) >  = 1 (Fig. 1). Using the Gene Ontology Reactome assay, we found that 13 mRNAs were enriched in the metabolic process of ATP and were subsequently involved in copper-iron homeostasis (Fig. 2A). We identified 139 mRNAs associated with Cu-related pathways from previous Gene Set Enrichment Analysis (GSEA) plots. Differentially expressed mRNAs were visualized in a volcano plot, with copper homeostasis-related genes highlighted (Fig. 2B). Cluster analysis of these genes is depicted in Fig. 2C, and further PPI analysis was used to explore the interactions among the DEGs related to copper homeostasis (Fig. 2D). With a minimum interactive score of 10 for PPI analysis, key hub genes, such as *SP1*, *CNND1*, *ALB*, *CDK1*, *CDKN2A*, *CASP3*, *PTEN*, *JUN*, *GSK3B*, *MDM2*, *AKT1*, *FOXO3*, *FOXO1*, *NFE2L2*, *PI3KCA*, and *SOD3*, were identified. The network comprising these genes is shown in Fig. 2E. Finally, Kaplan–Meier analysis was performed to explore the association between cuprotosis-related genes and BC patient prognosis, revealing that most did not show statistical significance, except for *PDHA1* (Sup Fig. 1).

### Enrichment analysis of copper homeostasis-related DEGs

Gene Ontology (GO), Reactome, and Kyoto Encyclopedia of Genes and Genomes (KEGG) pathway analyses were conducted to further elucidate the function of copper homeostasis-related mRNAs (chrmRNAs) in BC. Functional enrichment analysis revealed that the biological process (BP) was primarily enriched in detoxification, regulation of biological quality, cellular response, and stress response to copper ions (Sup Fig. 2A). Molecular function (MF) mainly comprised oxidoreductase activity, metal ion binding, cation binding, and transition metal ion binding (Sup Fig. 2B). In addition, the Golgi apparatus, early endosomes, the lumen of the endoplasmic reticulum, and extracellular exosomes were regular cellular components (CC) (Sup Fig. 2C). The differential expression of these genes is shown in the following reactome enrichment results: metallothionein binding, metal ion response, cellular response to chemical stress, intracellular signaling by second messengers, and RIP3 activation of AKT signaling (Sup Fig. 2D). The KEGG results indicated that mineral absorption, platinum drug resistance, endometrial cancer, ferroptosis, and cellular senescence ranked highest in these pathways (Sup Fig. 2E). Collectively, these findings suggest that copper metabolism and oxidative stress responses are integral to the pathophysiology of breast tumors.

### BRCA classification upon the copper homeostasis-related DEGs

To explore the relationship between the DEGs related to copper homeostasis and various breast cancer subtypes, we conducted a consensus cluster analysis of 605 breast cancer patients from the TCGA cohort. By increasing the clustering variable (*k*) between 1 and 6, we discovered the most significant correlation within groups, whereas intergroup correlations remained low when *k* = 4, suggesting that all samples could be divided into four clusters based on copper homeostasis-related DEGs (Sup Fig. 3A–C). The KM survival curve indicated that patients in Cluster 1 exhibited the highest survival probability (Sup Fig. 3D).

**Figure 3:**
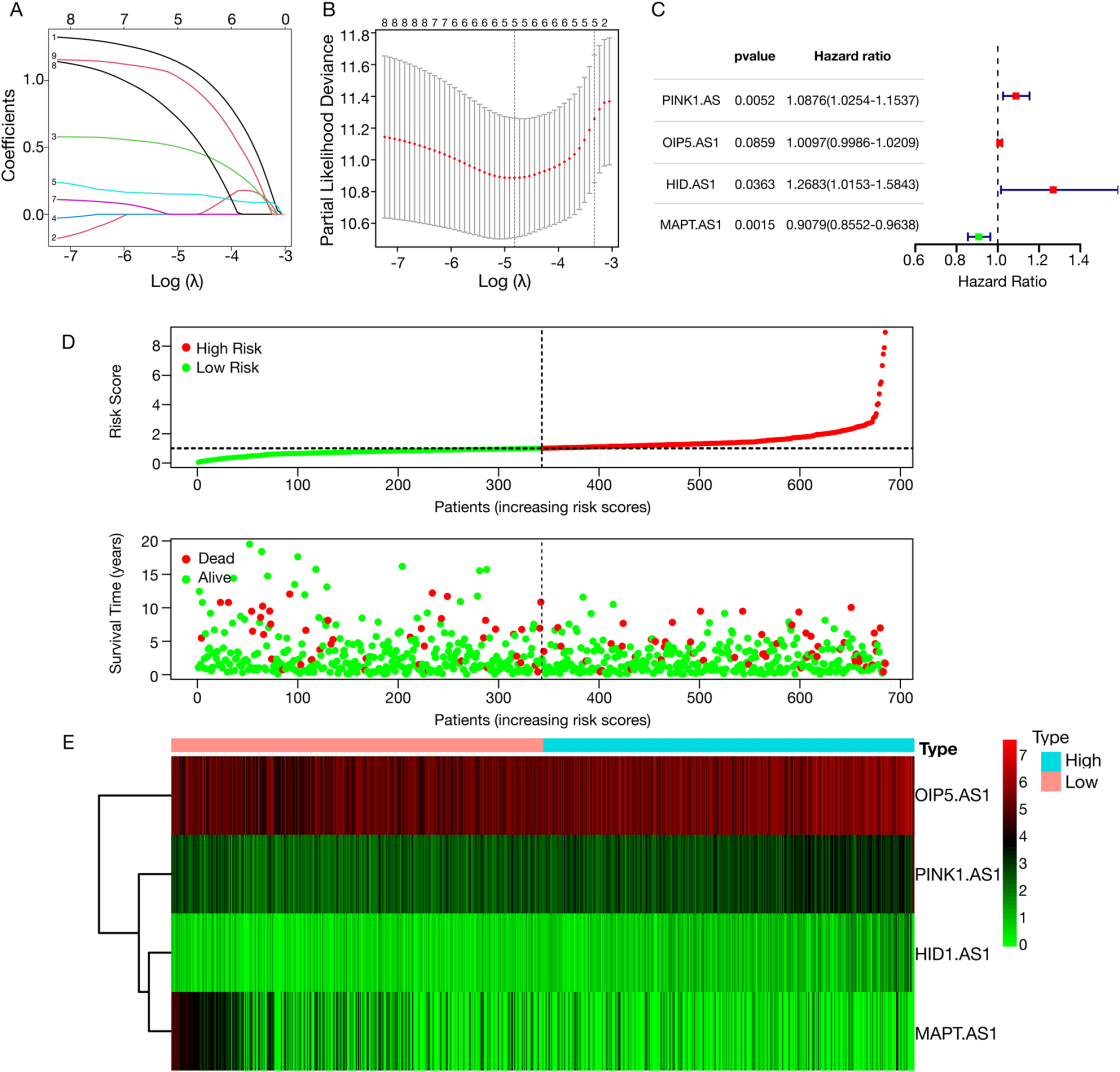
Establishment and verification of the risk assessment model. (**A**) ChrlncRNAs penalized by LASSO Cox regression analysis, with a coefficient profile plot generated against the log lambda sequence; (**B**) Tuning parameter (lambda) selection in the LASSO model employed tenfold cross-validation based on minimum criteria. Optimal values were identified by the minimum criteria and the one standard error of the minimum criteria (one-SE criteria), selecting a lambda value of 0.00048, log (lambda) − 3.32; (**C**) A forest plot illustrated the results of multivariate Cox analysis, indicating four chrlncRNAs associated with prognosis; (**D**) Risk score distribution of each BRCA sample, with green and red dots representing patients with low and high risk scores, respectively; (**E**) Scatter diagram depicting BRCA patient survival based on risk scores.

### Identification and validation of copper homeostasis-related long noncoding RNAs in BRCA patients

To determine whether the chrlncRNAs were associated with the chrmRNA mentioned above, *Pearson* correlation analysis was conducted with a standard of the absolute value of *Pearson* *R* > 0.4 and *p* < 0.001. This co-expression analysis led to the inclusion of 409 chrlncRNAs, as shown in Sup Table 1.

**Table 1:** Correlation between the risk score based on chrlncRNAs signature and clinicopathological features of 72 patients with primary breast cancer.

	Risk score		*P* value
	Low risk score	High risk score	
Age			0.073^a^
< 65	25	25	
65 ≥	6	16	
T stage			0.022^a^
1 + 2	45	17	
3 + 4	3	7	
N stage			0.006^a^
0	28	15	
1 + 2 + 3	9	20	
M stage			0.010^b^
0	37	29	
1	0	6	
AJCC stage			< 0.001^a^
0 + I + II	34	10	
III + IV	5	23	
Grading			0.006^a^
1 + 2	28	12	
3	12	20	
Ki67 (%)			< 0.001^c^
	21.56 ± 13.65	37.92 ± 21.92	
ER (%)			0.017^c^
	74.58 ± 34.17	51.97 ± 43.31	
PR (%)			0.025^c^
	57.86 ± 28.85	35.06 ± 39.34	
Pathological type			0.016^a^
HR^+^HER2^−^	27	22	
HER2^+^	10	4	
Triple negative	1	8	
HER2 expression			0.038^b^
HER2-0	4	10	
HER2-low	18	25	
HER2-positive	11	4	
Cancer embolus			< 0.001^a^
No	30	15	
Yes	7	20	
Perineural invasion			< 0.001^a^
No	60	2	
Yes	4	6	

^a^Pearson Chi-square Test.

^b^Fisher’s Exact Test.

^c^Single sample t-student test.

*AJCC* the American Joint Committee on Cancer, *HR* hormone receptor, *HER2* human epidermal growth factor receptor 2, *ER* estrogen receptor, *PR* progesterone receptor.

Univariate and multivariate Cox analyses were performed to further explore the chrlncRNAs significantly linked to the prognosis of BC patients, and four chrlncRNAs were screened, including PINK1.AS, OIP5.AS1, HID.AS1, and MAPT.AS1. Ten-fold cross-validation of the LASSO regression was used to obtain the best lambda value from the deviance of the least partial likelihood (Fig. 3A). The four chrlncRNAs showed a significant relationship with lambda values (Fig. 3B). To determine the prognostic significance of chrlncRNAs in patients with BC, we conducted a multivariate Cox proportional hazard regression analysis for the four genes (Fig. 3C). Based on the risk score, the gene signature model = (1.0876 × PINK1.AS exp.) + (1.0097 × OIP5.AS1 exp.) + (1.2683 × HID.AS1 exp.) + (− 0.9079 × MAPT.AS1 exp.). To validate the prognostic model, we calculated the risk scores of 605 patients with breast cancer and divided them into low- and high-risk groups based on the median score. The high-risk group exhibited a higher number of deaths and shorter survival time than the low-risk group (Fig. 3D). A heatmap of the four prognostic lncRNAs is shown in Fig. 3E. The expression of OIP5.AS1, HID1.AS1, and PINK1.AS1 was upregulated, whereas that of MAPT.AS1 was downregulated in the high-risk-score group.

**Figure 4:**
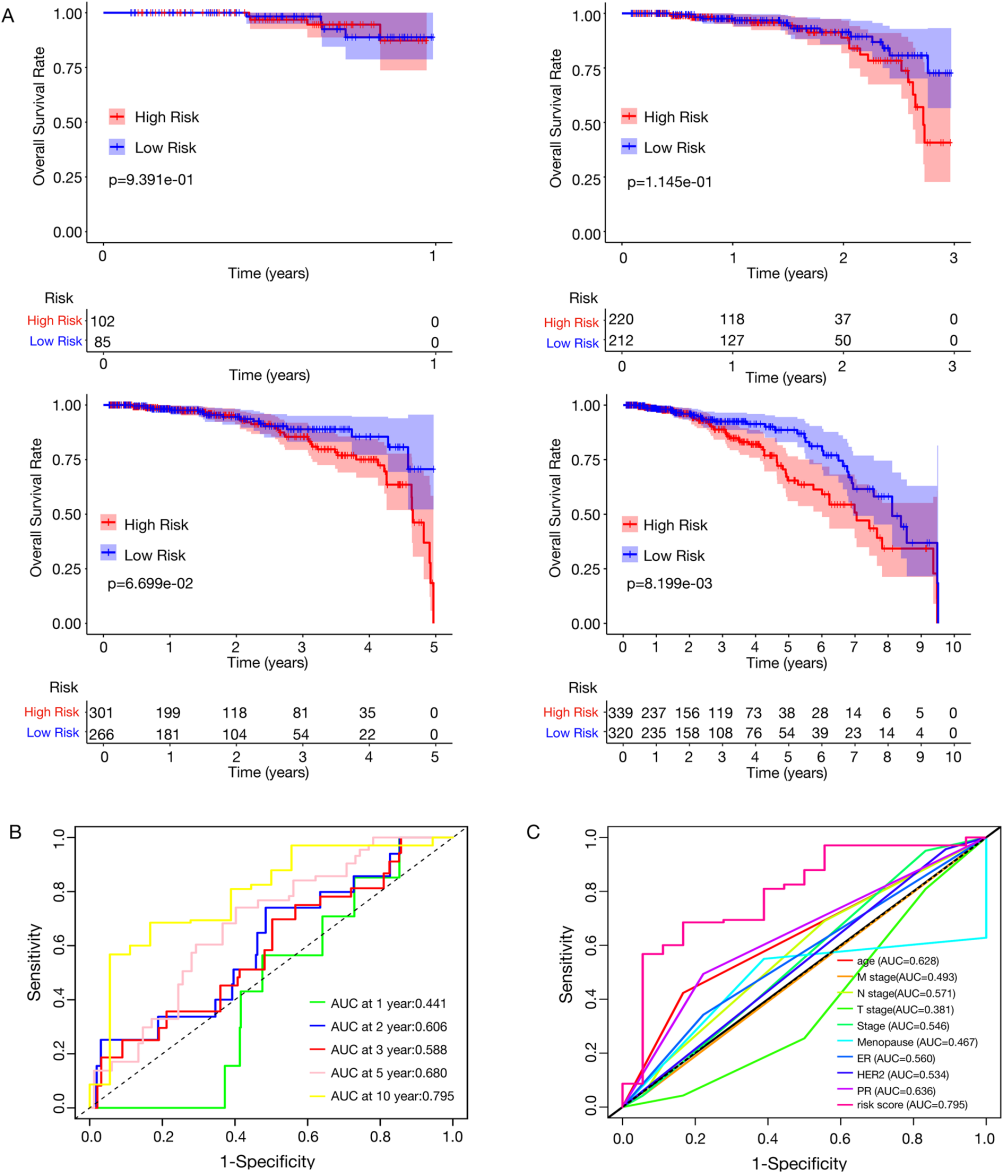
Survival analysis of the four copper homeostasis-related genes signature in the TCGA-BRCA cohort. (**A**) Kaplan–Meier survival curves categorized breast cancer patients from the TCGA database into high or low-risk groups at 1, 3, 5, and 10-year intervals. Overall survival (OS) differences between groups were evaluated using a two-sided log-rank test, revealing that higher risk scores are significantly associated with worse OS over ten years (*p* = 0.008); (**B**) Receiver Operating Characteristic (ROC) curve analysis of the four-gene copper homeostasis-related signature over 1, 2, 3, 5, and 10 years; (**C**) ROC curve analysis comparing the four-gene copper homeostasis-related signature to clinicopathological characteristics over 10 years.

Kaplan–Meier survival analysis indicated that patients with lower risk scores based on copper homeostasis-related lncRNAs had significantly better prognostic outcomes, with the disparity in survival rates between the groups widening over time (Fig. 4A). Additionally, Receiver Operating Characteristic (ROC) curves demonstrated the prognostic prediction capabilities of chrlncRNA gene signatures, with the area under the curve (AUC) for these chrlncRNAs increasing from 0.441 at one year to 0.795 at ten years (Fig. 4B). This indicates that the lncRNA-based risk score model may offer more precise prognostic predictions within ten years compared to traditional pathological features. The AUC of the risk score was 0.795 in predicting the overall survival of BC patients, which was superior to that of clinicopathological variables, including age, M stage, N stage, T stage, stage, menopausal status, ER expression, HER2 expression, and PR expression (Fig. 4C).

The Principal Component Analysis (PCA) further demonstrated the ability to differentiate patients into distinct risk groups. The 3-dimentional PCA plots showed clear segregation between the two groups of BC patients, offering a more nuanced understanding of risk than traditional gene- or RNA-based models, which were judged based on whole genes (all genes), whole RNA genes (all RNA), chrmRNAs, or chrlncRNAs (Sup Fig. 4).

### Correlation between expression of copper homeostasis-related mRNAs and lncRNAs in BRCA patients

We further visualized the chrmRNAs-chrlncRNAs network using the Sankey diagram with the *R Pearson* correlation package (Sup Fig. 5A). The co-expression network contained 20 pairs of chrmRNAs-chrlncRNAs related to copper homeostasis. Notably, *OIP5.AS1 *was co-expressed with several key genes, including* SP1*, *APC*, *XIAP*, *PTEN*, *ADAM10*, *ATP7A*, *GSK3β*, *MTF1*, *NFE2L2*, and *ADNP* (Sup Fig. 5B). *HID1.AS1* was positively associated with eight copper homeostasis-related genes: *SNCA*, *AOC2*, *PTEN*, *SOD3*, *F8*, *AQP1*, *FOXO1*, and *AOC3* (Sup Fig. 5C). Furthermore, *PINK1.AS* was found to be co-expressed with *MTF1* and *MAPT.AS1* exhibited a positive relationship with *MAPT* (Sup Fig. 5D). These results highlight the involvement of chrlncRNAs in the oxidative stress response in breast cancer, as exemplified by the association of ROS-related genes, such as *NFE2L2,* with copper homeostasis.

**Figure 5:**
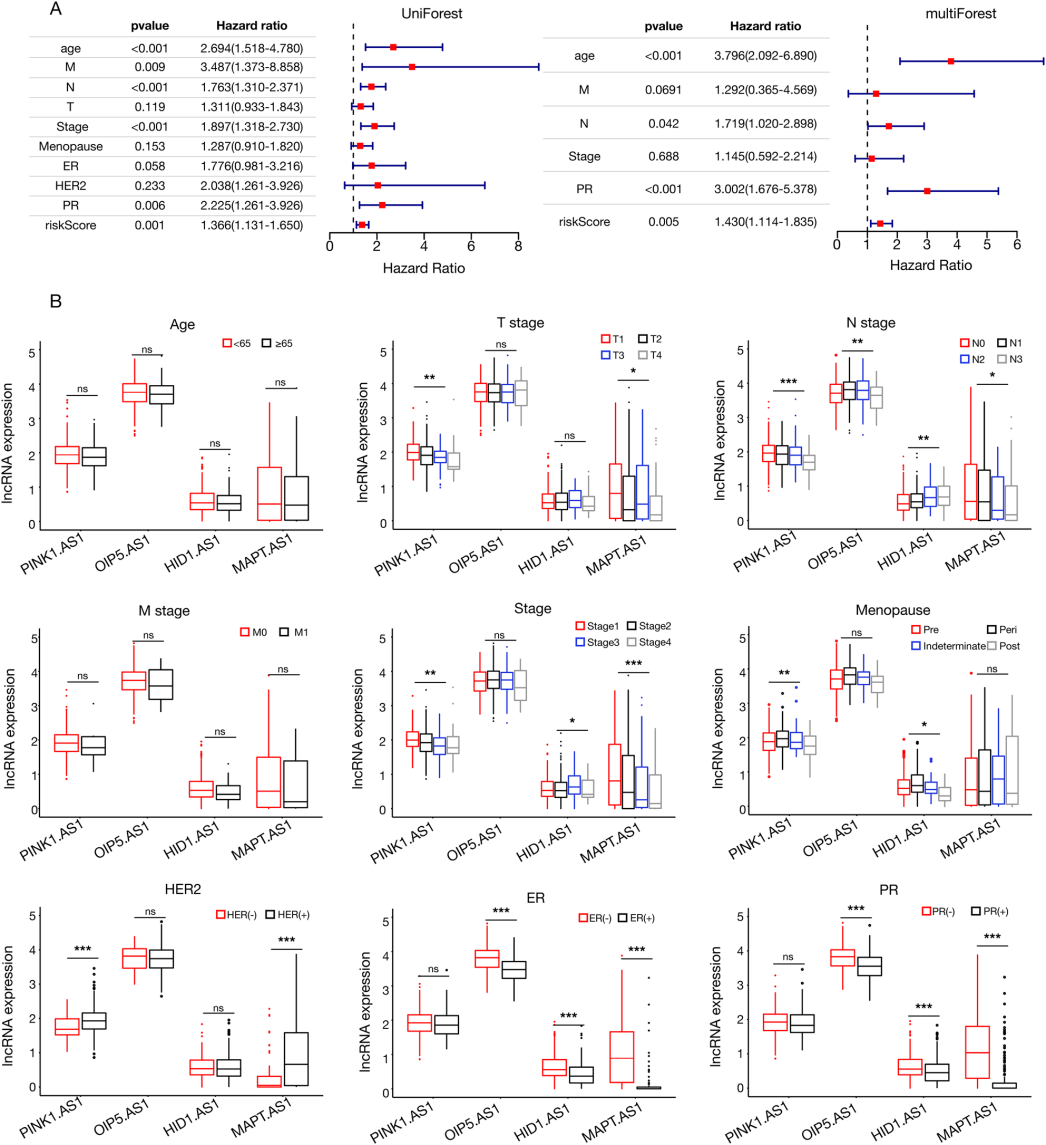
Association of clinical-pathological profiles with chrlncRNAs in breast cancer patients. (**A**) Univariate and multivariate Cox analysis of the TCGA BRCA cohort; (**B**) Boxplots illustrating the correlation of chrlncRNAs with clinicopathological characteristics. HER2: human epidermal growth factor receptor 2, ER: estrogen receptor, PR: progesterone receptor, *:* p* < 0.05, **: *p* < 0.01, ***: *p* < 0.001, ns: none significance.

### Correlation between the chrlncRNAs gene signature and the clinical characteristics in BC patients

A univariate Cox progression analysis was initially performed to verify the predictive reliability of the chrlncRNAs gene signature model for the clinical outcomes of patients with breast cancer. This analysis highlighted significant correlations between age, M stage, N stage, overall stage, PR status, risk score, and the overall survival of patients with breast cancer. Subsequent multivariate Cox regression analysis further confirmed the significant prognostic relationship between age, N stage, PR status, and risk score (Fig. 5A). We examined the association between these four chrlncRNAs and various clinical features. Figure 5B shows that PINK1.AS1 correlated with the T classifier, N classifier, overall stage, menopausal status, and HER2 expression. In contrast, OIP5.AS1 showed a significant relationship with three characteristics: N stage, ER, and PR status. N stage, menopausal status, ER status, and PR status were all closely associated with HID1.AS1 expression. MAPT.AS1 was significantly correlated with T stage, N stage, HER2 stage, ER status, and PR status.

Additionally, a nomogram incorporating clinicopathological characteristics and risk scores was developed to enhance the prediction of overall survival for patients with BC. Sup Fig. 6A shows that the nomogram model and calibration curve could predict the 1-, 2-, 3-, 5-, and 10-year prognoses, with the calibration curves at ten years, demonstrating the accuracy of the model by aligning the predicted survival rates with the actual overall survival rates of breast cancer patients (Sup Fig. 6B).

**Figure 6:**
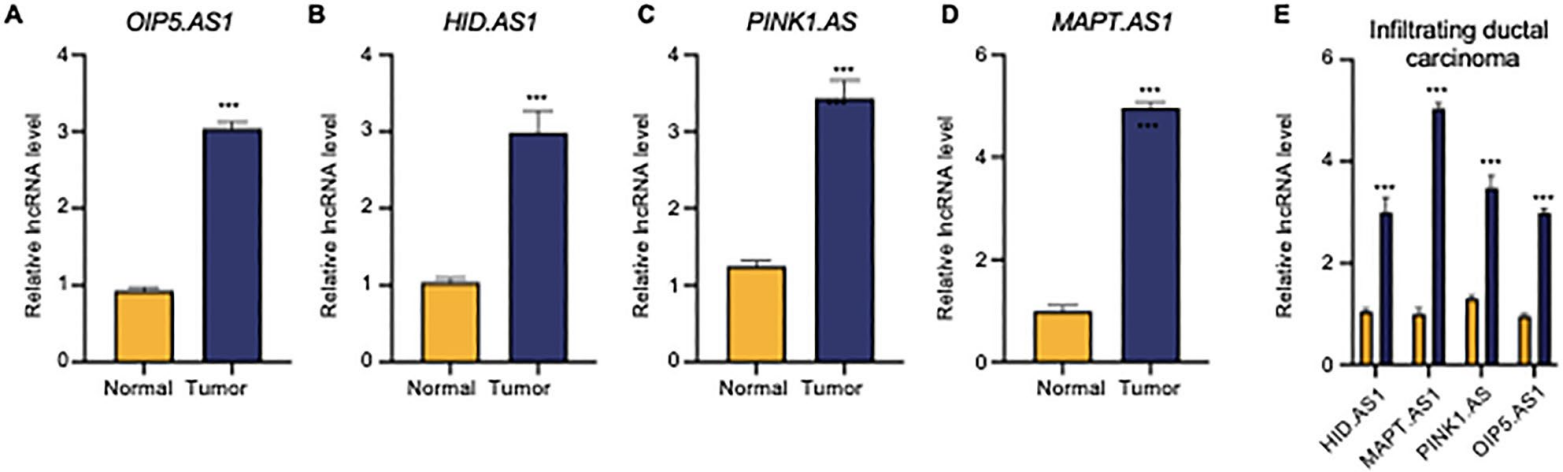
Quantitative analysis of four chrlncRNAs expression in normal and tumor tissues by using quantitative real-time polymerase chain reaction (qRT-PCR). (**A**–**D**) Relative expression level of chrlncRNAs in normal and tumor tissues from 72 patients with primary breast cancer. (**E**) Relative expression of four chrlncRNAs in normal versus tumor tissues in the subtype of ductal infiltrating carcinoma. Expression differences between the two groups were assessed using single sample t-student test. **: *p* < 0.01, ***: *p* < 0.001, ns: none significance.

### Machine learning algorithms detecting the association between the gene signature and the sensitivity of anticancer drugs

We applied the "pRRophetic" R package^14^ and machine learning alogorithms to detect the association between the gene signature and the sensitivity of anticancer drugs. This method successfully identified 24 anticancer drugs with a significant correlation between their sensitivity (measured by inhibitory concentration, IC_50_) and our model. Among these 24 drugs, patients with high-risk scores showed higher sensitivity with 14 anticancer drugs: GNF.2 (*p* < 0.001), GSK269962A (*p* < 0.001) AZD8055 (*p* < 0.001), Rapamycin (*p* < 0.001), Bortezomib (*p* = 0.002), Lenalidomide (*p* = 0.009), FTI.277 (*p* < 0.001), GDC.0449 (*p* < 0.001), PD.0332991 (*p *= 0.001), Thapsigargin (*p* = 0.014), JNK.Inhibitor.VIII (*p* = 0.016), Parthenolide (*p* = 0.014), Gefitinib (*p* < 0.001), MS.275 (*p* = 0.007), while patients with low-risk scores showed higher sensitivity with 10 anticancer drugs: Camptothecin (*p* < 0.001), SB.216763 (*p* < 0.001), Etoposide (*p* < 0.001), FH535 (*p* < 0.001), Tipifarnib (*p* < 0.001), JNK.9L (*p* < 0.001), Vinblastine (*p* < 0.001), Mitomycin.C (*p* < 0.001), X17.AAG (*p* = 0.038), NSC.87877 (*p* < 0.001) (Sup Fig. 7). Notably, Bortezomib is currently being investigated in a phase II clinical trial as a monotherapy for patients with metastatic breast cancer^15^. Furthermore, a phase II trial has assessed the combination of gefitinib and tamoxifen in patients with hormone receptor-positive metastatic breast cancer^16^.

### Validations of the expression levels of the chrlncRNAs and gene signature model in clinical specimens

To validate our preclinical findings, we analyzed clinical samples from 72 chemotherapy-naïve patients with breast cancer, including primary breast cancer tissues and matched adjacent normal tissues. Comprehensive clinical data was available for all participants (Sup Table 2). We assessed the expression of PINK1.AS, OIP5.AS1, HID.AS1, and MAPT.AS1 in tumor pairs using qRT-PCR, which revealed a marked increase in the expression of these chrlncRNAs in tumor tissues compared to adjacent tissues (Fig. 6A–D). DNA gel electrophoresis was performed to evaluate qPCR products (Sup Fig. 8). Notably, the expression of PINK1.AS, OIP5.AS1, HID.AS1, and MAPT.AS1 was statistically higher in the infiltrating ductal carcinoma subtype (Fig. 6E). By applying our gene signature model, we calculated risk scores for all patients and categorized them into low- and high-risk groups based on the median score (Table 1). In the high-risk subgroup, patients were significantly associated with the clinicopathological parameters of advanced T (*p* = 0.022), M (*p* = 0.010), and N stage (*p* = 0.006), advanced grading (*p* = 0.006), advanced AJCC stage (*p* < 0.001), higher ki-67 expression (*p* < 0.001), lower ER (*p* = 0.017), PR expression (*p* = 0.025), cancer emboli (*p* < 0.001), and perineural invasion (*p* < 0.001). The risk score was also correlated with the pathological type and HER2 expression level, indicating its potential utility in devising targeted therapies for breast cancer. These clinical observations reinforce the link between our gene signature model and the malignant traits of breast cancer, suggesting a pivotal role for chrlncRNAs in disease initiation and progression.

## Discussion

Redox homeostasis is increasingly recognized as a critical target for anticancer strategies, with Cu playing a vital role because of its variable redox states^17^. The involvement of copper in numerous metabolic reactions necessitates a careful balance between its uptake and distribution, impacting processes such as energy production and iron transport^18^. Although cancer cells require high Cu levels for hypermetabolism and proliferation, the precise implications of Cu in oncogenesis and cancer progression are not fully understood^19^. In this study, we extensively analyzed copper homeostasis-related gene profiles in patients with BC and developed a novel prognostic lncRNAs signature consisting of PINK1.AS, OIP5.AS1, HID.AS1, and MAPT.AS1 using LASSO Cox regression. This signature was notably correlated with clinicopathological features and demonstrated predictive ability for BC patient prognosis. Additionally, the expression of these chrlncRNAs was elevated in tumor tissues compared to adjacent normal tissues in our cohort of 72 patients with BC. Moreover, the high-risk group had more adverse clinical characteristics than the low-risk group. Collectively, these findings indicate the efficacy of the chrlncRNA-related signature for prognosticating BC outcomes, thereby providing valuable insights for future clinical applications.

Copper and its biological molecules are pivotal in redox reactions because they interact directly with oxygen to generate free radicals. Imbalances in copper homeostasis can lead to intracellular toxicity and are implicated in conditions such as Wilson and Menkes disease, in which genetic mutations cause copper overload or deficiency^18^. Recent studies have reported elevated serum Cu levels in various cancers, including breast cancer, with notably higher levels in cancer patients compared to controls^20^. Additionally, an increased Cu/Zn ratio in plasma and urine has been associated with increased breast cancer risk and poor prognosis, independent of ER/PR/HER2 status^21^. Recent research has shown that excessive Cu can induce a novel PCD known as cuproptosis^6^. During cuproptosis, copper ions bind to TCA cycle components, leading to lipid acylation, proteotoxic stress, and cell death^6^. However, cuproptosis-related genes, including FDX1, LIPT1, LIAS, DLD, DLAT, and PDHB, failed to reach statistical differences by Kaplan–Meier analysis in our study and were unable to build a prognostic survival model, indicating the need for a more nuanced understanding of the roles of these genes in breast cancer. Targeting Cu imbalances and Cu-dependent pathways is a promising therapeutic approach. For example, the copper chaperone inhibitor DCAC50 disrupts copper homeostasis and induces apoptosis in triple-negative breast cancer cells^22^. This suggests that gene signatures associated with copper homeostasis could serve as valuable indicators for targeted therapy in patients with breast cancer, highlighting the potential for novel treatments that exploit the unique biological role of copper.

Notably, the significant prior research has investigated the association between cuproptosis and breast cancer^23–28^. For example, one study identified a risk score derived from nine cuproptosis-related lncRNAs (LRRC8C-DT, TDRKH-AS1, SAMMSON, SIAH2-AS1, WDFY3-AS2, LINC00393, ARHGAP28-AS1, PCAT18, LINC01711), establishing it as an independent prognostic factor for BC and a potential indicator for immunotherapy response^23^. Similarly, a separate study presented a cuproptosis-related gene signature (involving TNFRSF18, SLC1A1, among others) to assess patient outcomes and the tumor immune environment, although it lacked clinical sample validation^25^. Pan et al. identified and validated 10 cuproptosis-related lncRNAs in 12 BC patients and cell lines^26^, while Huang et al. concentrated on cuproptosis-related genes, identifying PDHA1 as an independent BC prognostic biomarker, validated in cell lines and a 30-sample tissue microarray^27^. However, current research focusing on lncRNAs related to copper homeostasis, instead of those associated with cuproptosis and breast cancer, is still largely uncharted. Additionally, there is a notable gap in the comprehensive validation of a large clinical cohort, which is essential to confirm both the stability and the diagnostic utility of the proposed model.

The genes (PINK1.AS, OIP5.AS1, HID.AS1, and MAPT.AS1) identified in our study all belonged to antisense lncRNAs that knocked down the complements of the endogenous sense homolog and comprised a large proportion of the long noncoding transcriptome^29^. Notably, OIP5.AS1 is recognized for its oncogenic role in various cancers and its involvement in cellular proliferation^30^. OIP5. AS1 knockdown inhibits breast cancer cell proliferation and invasion by affecting the epithelial-mesenchymal transition (EMT) process, whereas overexpression enhances these malignancies^31^. Our study's chrmRNAs-chrlncRNAs network analysis indicated associations of OIP5.AS1 with key pathways and proteins like GSK3β, a crucial regulatory kinase interacting with signaling pathways such as Wnt/β-catenin and PI3K/AKT signaling. In gastric carcinoma, OIP5.AS1 regulates PI3K/AKT and Wnt/β-catenin pathways in a High Mobility Group AT-Hook 2 (HMGA2)-dependent way^32^. Whether GSK3β is involved in the interaction between OIP5.AS1 and these pathways are worthy of meditation. We found that OPI5.AS1 was also tightly co-expressed with the critical copper transporter ATPase Copper Transporting Alpha (ATP7A)^33^, raising the possibility that OIP5.AS1 might interfere with ATPA-mediated copper export. HID.AS1, another gene in our signature, is a known prognostic marker in breast cancer^34^ and was found to be positively associated with FOXO1. This gene promotes copper-related antioxidant protein expression related to copper^35^. MAPT.AS1 transcribed from the antisense strand of the Microtubule Associated Protein Tau (MAPT) promoter region, exhibiting conflicting roles in breast cancer. Overexpression of MAPT.AS1 promoted malignancy through activation of the Wnt/β-catenin signal^36^ and has also been correlated with the malignant phenotype and chemotherapy resistance of ER-negative breast cancer^37^. In contrast, elevated MAPT.AS1 expression was observed in patients with BC with longer survival times^38^, which is consistent with our results. Notably, the gene signature constructed by our group emphasized the interaction of the gene network rather than the biological impact of an individual gene. High plasma PINK1.AS levels in small cell lung cancer patients predicted distant metastasis^39^. In addition, PINK1.AS promoted gastric cancer progression by sponging miR-200a to negatively regulate the expression of G Protein Subunit Alpha I1 (Gαi1) expression^40^. To date, research on PINK1.AS in breast cancer has not been conducted. Due to the close correlation between PINK.AS and MTF1, we hypothesized that PINK.AS disrupts metal homeostasis in response to copper by indirectly controlling the expression of Metal Regulatory Transcription Factor 1 (MTF1). Collectively, these findings underscore the complex role of copper homeostasis-related lncRNAs in cancer development and prognosis. However, the detailed mechanisms by which these lncRNAs influence breast cancer require further investigation to fully understand their potential as therapeutic targets and prognostic markers.

Utilizing the "pRRophetic" package and machine learning algorithms, we identified 24 anticancer drugs with a significant correlation between their sensitivity (IC_50_) and our predictive model. Among these, 14 drugs exhibited lower IC_50_ values and higher sensitivity in the high-risk group, including Bortezomib, Gefinib, Lenalidomide, Rapamycin, Thapsigargin, and Parthenolide. Notably, Bortezomib is currently undergoing a phase II clinical trial as a single agent for patients with metastatic breast cancer^15^. Additionally, a phase II trial has evaluated the combination of gefitinib and tamoxifen in patients with hormone receptor-positive metastatic breast cancer^16^. Our analysis of drug sensitivity in both high- and low-risk groups facilitates the identification of drugs that may yield better treatment outcomes for high-risk patients, potentially extending their survival and enhancing their prognosis. Moreover, the highly sensitive anti-breast cancer drugs identified in our study provide substantial big data support for the integration of targeted drugs into clinical trials.

In our study, clinical sample analysis revealed higher expression levels of chrlncRNAs in tumors than in the adjacent normal tissues, as determined by qRT-PCR. By integrating lncRNA expression with our gene signature model, we calculated the risk scores for each patient and divided them into low- and high-risk groups. The high-risk group demonstrated a correlation with more severe clinical manifestations in breast cancer, supporting our initial findings from the TCGA cohort analysis. Moreover, high-risk scores were associated with invasive tumor behavior, including cancer emboli and perineural invasion. Recent advancements in breast cancer treatment have identified HER2-low breast cancer as a targetable subtype, particularly with the emergence of anti-HER2-antibody drug conjugates^41^. Despite the challenges of the traditional HER2 status classification, our study found a significant association between high-risk scores and varying levels of HER2 expression in our 72-patient cohort. This underscores the potential utility of our risk signature in identifying candidates for potent anti-HER2 antibody–drug conjugates (ADCs), indicating a promising direction for personalized BC treatment strategies.

However, this study has some limitations. Firstly, the patient cohort lacked essential prognostic data. To ascertain the utility of our predictive signature, we need access to additional databases or comprehensive follow-up data. Secondly, the specific criteria for surgical eligibility might limit the applicability of our gene signature model, particularly in patients with advanced BC. Although our model shows promise in predicting outcomes for primary breast cancer patients, it falls short in addressing the heterogeneity of breast cancer subtypes. A more nuanced, subtype-specific analysis is necessary to fully understand the influence and potential impact of lncRNAs on the subtypes^42^. Finally, the mechanism of action of copper homeostasis-related lncRNAs in BC remains unclear and requires further experimental validation. Nevertheless, our study is also promising in raising great hope for exploring the role of copper hemostasis-related lncRNAs in breast cancer and can help assess the clinical outcome based on this gene signature model.

In summary, we identified a novel model of four copper homeostasis-related lncRNAs using the TCGA BRCA cohort. On one hand, lncRNAs, as non-coding RNAs, can alter the invasive and metastatic abilities of tumors, thus affecting patient prognosis. The prognostic risk score showed excellent performance in the prediction of the survival of patients with breast cancer, and the nomogram model could help make a prognosis assessment as a reference. Additionally, our drug sensitivity analysis enhances the clinical applicability of our model by aiding the transition of effective anticancer drugs from research to clinical practice. In our cohort, we examined chrlncRNAs expression between tumor and normal tissues and demonstrated the value of the chrlncRNAs gene signature in predicting the progression of primary breast cancer. Our results would provide novel insights for developing new targeted therapies for copper homeostasis for early cancer diagnosis and treatment.

## Methods

### Clinical samples

This study included 72 patients with breast cancer, all of whom were confirmed by mammographic screening and pathological evaluation at the Department of Breast and Thyroid Surgery, Renmin Hospital, Wuhan University. Participants were included based on the following criteria: (1) age of 18 years or older; (2) a new diagnosis of breast cancer; and (3) eligibility for surgery with adjunct therapy. The exclusion criteria were: (1) comorbidities with other malignant tumors and infections; (2) cognitive or psychiatric disorders; and (3) distant metastasis and loss of surgical opportunity, except for bone metastases. All collected patient data, including clinical and molecular details, were systematically recorded and summarized in Sup Table 3. Biopsies were performed according to standard care protocols, and samples were immediately snap-frozen and preserved in liquid nitrogen for subsequent analysis. All methods were conducted according to the appropriate guidelines and regulations.

### Data collection and processing

The TCGA BRCA database serves as an extensive and carefully curated collection of molecular and clinical information on many patients with breast cancer. It included a wide array of patient samples covering different breast cancer subtypes, disease stages, and detailed clinical information. We chose the TCGA database to assess the clinical characteristics of breast cancer patients and analyze gene expression profiles in both cancerous and adjacent normal tissues. RNA sequencing data (112 normal tissues and 1085 tumor samples) were retrieved from the TCGA-BRCA database. After normalizing the original RNA-seq data to transcripts per million (TPM), differentially expressed genes (DEGs) were identified through the application of the "limma" package in R. Copper homeostasis and cuprotosis-related genes were selected from the Gene Set Enrichment Analysis (GSEA) pathway datasets following the guidelines from previous reports^6^ (Sup Table 4).

### Clustering of copper homeostasis-related regulators and signaling pathways

PPI network of copper homeostasis-related DEGs (chrDEGs) was constructed using the STRING database and visualized using Cytoscape version 3.9.1. Pearson’s correlation analysis was performed to elucidate the relationships between the different regulatory modules. To categorize the prognostic significance of the chrDEGs within these modules, we applied optimal* k*-means clustering using the ConsensusClusterPlus R package and divided them into distinct clusters.

Gene Ontology (GO) analysis containing biological process (BP), cellular component (CC), and molecular function (MF) was applied using the R package "Cluster Profiler", which was intended to elucidate the functional role of the chrDEGs. The biological pathways associated with chrDEGs were explored using Kyoto Encyclopedia of Genes and Genomes (KEGG) data^43–45^ via the DAVID and Reactome databases using the R package "ReactomePA".

### The development and validation of the copper homeostasis-related lncRNAs prognostic signature

The correlation between copper homeostasis-related mRNAs and lncRNAs was calculated using the "stats" R package. Under the correlation coefficient |R|> 0.4 and *p* < 0.001, we implemented significant co-expression of the related genes. To develop a prognostic lncRNAs signature for BC patients, we identified copper homeostasis-related lncRNAs (chrlncRNAs) and conducted survival analyses using Cox and LASSO regressions. This led to the establishment of the chrlncRNAs gene signature model. Patients were stratified into high-risk (≥ median) and low-risk (< median) groups based on the model's median risk score. Furthermore, the model was integrated with clinical parameters, including age, T stage, N stage, M stage, overall stage, menopausal status, and expression levels of ER, HER2, and PR. This integrated risk score prognostic model was then analyzed and validated through both univariate and multivariate Cox regression analyses using the Kaplan–Meier "survival" R package. The model's efficacy was assessed using time-dependent receiver operating characteristic (ROC) curves, with clinical feature ROC curves generated by the "survivalROC" R package. The "survminer" R package was utilized to create Kaplan–Meier survival curves, comparing the survival rates between high-risk and low-risk groups. Finally, PCA analysis was used to further validate the prognostic accuracy of the lncRNAs model.

### Nomogram and calibration curves for evaluating the risk of breast cancer patients

We devised a hybrid nomogram model to facilitate a clinically viable method for predicting overall survival in breast cancer patients. This model integrates independent prognostic factors such as risk score, sex, age, TNM classification, stage, grade, menopausal status, and expression of ER, PR, and HER2 using the "RMS" R package. Calibration and discrimination are typically employed as primary metrics in model evaluation. In our study, calibration curves were utilized to graphically compare the nomogram-predicted probabilities with the observed rates, where the ideal predictions were aligned with the 45° line.

### Screening of anticancer drugs

We utilized the "pRRophetic" R package and machine learning algorithms^14^ to screen out 24 anticancer drugs that showed a significant relationship with the model based on their sensitivity (IC_50_). The significance threshold for the *p*-value was set at 0.05, and the anticancer drug sensitivity database used was CPG 2014, which is incorporated in the "pRRophetic" R package.

### Quantitative real-time PCR (qRT-PCR)

Total RNA was extracted from breast cancer and adjacent normal tissues using NucleoZOL reagent (Takara, Shiga, Japan) according to the manufacturer's protocol. Total RNA was quantified in duplicate using a Nanodrop ^ND-2000^ spectrophotometer (Thermo Fisher Scientific, MA, USA). The extracted RNA was then reverse-transcribed into cDNA in a 20μL reaction volume (Vazyme, China). The primers were synthesized by Sangon Biotech (Shanghai, China). The primers for four chrlncRNAs were used as follows: PINK1.AS (forward: 5′ CGGAAATGCCTCCAGTCTCT 3′, reverse: 5′ TTCCTCGCATCTCCTGTTCC 3′), OIP5.AS1 (forward: 5′ CTGAAGCCGCTCTATGGGTT 3′, reverse: 5′ CCCAGGAAACCAGTGGAGAA 3′), HID.AS1 (forward: 5′ CAACCAGGAACTCCCAAGTGA 3′, reverse: 5′ AGGAGAATGCCCATCTGAGT 3′), MAPT.AS1 (forward: 5′ CAGGCCAGGAGTCAGAAACAA 3′, reverse: 5′ CCTGGGCTACTGTTCCACAT 3′) and 18S rRNA (forward: 5′ GGAGTATGGTTGCAAAGCTGA 3′, reverse: 5′ ATCTGTCAATCCTGTCCGTGT 3′). Quantitative PCR was performed using the SYBR Green PCR reagent on a Light Cycler 480 II (Roche, Germany). Final data quantification was performed using the 2^−ΔΔCt^ method. For testing the qRT-PCR success, agarose gel electrophoresis was performed according to manufacter’s protocols^46^. In brief, a 2% agarose gel was prepared by dissolving agarose powder in Tris/Acetic /EDTA (TAE) buffer in an Erlenmeyer flask. The flask was covered to prevent evaporation and heated to ensure complete dissolving of the agarose powder. After boiling, Red Nucleic Acid Gel Stain (MedChemExpress, USA) was added and mixed thoroughly with the gel prior to casting it into the tray. After the gel had set, qRT-PCR products were mixed with loading dye and applied to the gel. Electrophoresis was run at 150 V for 50 min.

### Statistical analysis

The "Limma", "Survival", and "Surminer" R packages were used for data analysis. *Wilcox test* and unpaired *Student’s t-test* were used to compare non-normally and normally distributed expression variables, respectively. *Spearman correlation* was used to test the correlation between copper homeostasis-related mRNAs and lncRNAs; *p* < 0.05 in the results was considered statistically significant. *Pearson’s chi-square* test was used to differentiate clinical manifestations between patients categorized into low- and high-risk score groups. All statistical methods and values are listed in Sup Table 5.

### Ethical approval

Ethical approval was obtained from the Institutional Research Ethics Committee of Renmin Hospital, Wuhan University (No. WDRY2022-K002). Before commencing the research, informed consent was obtained from all participants. The consent process entailed a comprehensive explanation of the study's aims, methods, risks, and benefits, allowing participants to make informed choices. Written consent was obtained from each participant, adhering to the Declaration of Helsinki and ensuring voluntary engagement and rights protection. Clinical and molecular data were anonymized and assigned unique identifiers, with access restricted to authorized research team members.

### Supplementary Information

Supplementary Information 1.

Supplementary Information 2.

Supplementary Information 3.

Supplementary Information 4.

Supplementary Information 5.

Supplementary Information 6.

Supplementary Information 7.

Supplementary Information 8.

Supplementary Information 9.

Supplementary Information 10.

Supplementary Information 11.

Supplementary Information 12.

Supplementary Information 13.

Supplementary Information 14.

## Data Availability

In this study, we utilized publicly available gene expression datasets, specifically from the TCGA BRCA cohort, which are accessible at https://portal.gdc.cancer.gov/. For validation purposes, 72 pairs of primary breast cancer tissues and matched adjacent tissues were obtained from chemo-naïve breast cancer patients. These validation data were sourced from the Department of Breast and Thyroid Surgery, Renmin Hospital at Wuhan University. While these specific data are not publicly accessible due to restrictions and were used under license for this study, they are available from the authors upon reasonable request and with permission from the Department of Breast and Thyroid Surgery, Renmin Hospital at Wuhan University.
