# Supplementary material for: Identification and validation of a copper homeostasis-related gene signature for the predicting prognosis of breast cancer patients via integrated bioinformatics analysis
Source: Sci Rep. 2024 Feb 7;14:3141. doi: 10.1038/s41598-024-53560-9 (PMC10850146; doi:10.1038/s41598-024-53560-9)
Supplement: Supplementary file 1 — Supplementary Information 1. [file 41598_2024_53560_MOESM1_ESM.docx]

**Sup Fig. Legend:**

**Sup Fig. 1:** Overall survival analysis of ten cuprptosis-related genes in BC patients with low or high-expression group and expression of ten cuprptosis-related genes in BRCA patients. **(A)** Kaplan-Meier survival analysis of ten cuproptosis-related genes in breast cancer patients categorized into high or low-expression groups over 10 years. Overall survival (OS) differences between the groups were assessed using a two-side log-rank test. It was concluded that PDHA1 is significantly associated with worse OS over ten years among the cuproptosis genes (*p* < 0.0001); **(B)** Volcano Plot highlighting ten cuproptosis-related genes in BRCA patients.

**Sup Fig. 2:** Gene Ontology, Reactome, and KEGG enrichment analysis of the copper homeostasis-related DEGs. **(A-C)** Ridgeline plots of biological process (BP), molecular function (MF), and cell component (CC) analyses, respectively; **(D)** Ridgeline plots illustrating Reactome pathway analysis; **(E)** Chord Diagram depicting KEGG pathway analysis.

**Sup Fig. 3:** Breast cancer classification pattern based on copper homeostasis-related differentially expressed genes (DEGs). **(A-C)** Classification of 605 breast cancer (BC) patients into four distinct groups *(k*=4) within the TCGA BRCA cohort; **(D)** Kaplan-Meier analysis illustrating the overall survival (OS) curves for the four clusters. OS differences between the clusters were assessed using a two-sided log-rank test.

**Sup Fig. 4:** Three-Dimensional principal component analysis (PCA) of breast cancer patients from the TCGA Cohort, categorized by various expression profiles. **(A)** Whole gene expression; **(B)** All RNA expression; **(C)** Copper homeostasis-related long non-coding RNA (chrLncRNA) expression; **(D)** Copper homeostasis-related mRNA (chrmRNA) expression; **(E)** Risk model categorization into low and high-risk groups based on the gene signature.

**Sup Fig. 5:** Correlation between chrmRNA and chrlncRNA. **(A)** A Sankey diagram showing the regulatory network between chrmRNA and chrlncRNA; **(B-D)** Scatter plots depicting the correlation between chrmRNA with chrlncRNA, assessed using the *Pearson* correlation test.

**Sup Fig. 6:** Nomogram model and calibration curves based on risk score signature and prognosis-related clinicopathological indicators. **(A)** Nomograms integrating a four-chrlncRNA signature with clinical characteristics to predict 1-, 2-, 3-, 5-, and 10-year survival probabilities for BC patients; **(B)** Calibration curve for each model demonstrating agreement with 10-year outcomes. Model performance is indicated by alignment with the 45-degree line, representing perfect prediction.

**Sup Fig. 7:** The sensitivity of anticancer drugs. By using pRRophetic R package and machine learning, 24 anticancer drugs whose sensitivity (IC50) was significantly related to the model were screened out. The threshold of p-value was 0.05, and the anticancer drug sensitivity database used was CPG 2014.

**Sup Fig. 8:** Agarose gels post quantitative qPCR for assessing reproducibility of gene expression. Samples were organized by increasing brightness as analyzed by ImageJ. **(A-D)** Original agarose gels depicting the expression of OIP5.AS1, HID.AS1, PINK.AS1, and MAPT.AS1, respectively, between tumor and adjacent normal tissues from 72 patients with primary breast cancer, with background subtraction. Low-molecular-weight DNA ladder (LMW: M1100, Servicebio, China) included for reference.

**Sup Table 1:** Correlations between chmRNA and chrlncRNA with the standard of coefficient index >0.4.

**Sup Table 2:** Summary of clinical characteristics obtained from 72 patients with primary breast cancer.

**Sup Table 3:** Specific clinical characteristics obtained from 72 patients with primary breast cancer.

**Sup Table 4:** The identified 139 copper homeostasis-related genes signature and 10 cuproptosis-related genes.

**Sup Table 5:** Summary of statistical method and statistical value.
