# Supplementary figures and images for: Identification and validation of a copper homeostasis-related gene signature for the predicting prognosis of breast cancer patients via integrated bioinformatics analysis
Source: Sci Rep. 2024 Feb 7;14:3141. doi: 10.1038/s41598-024-53560-9 (PMC10850146; doi:10.1038/s41598-024-53560-9)

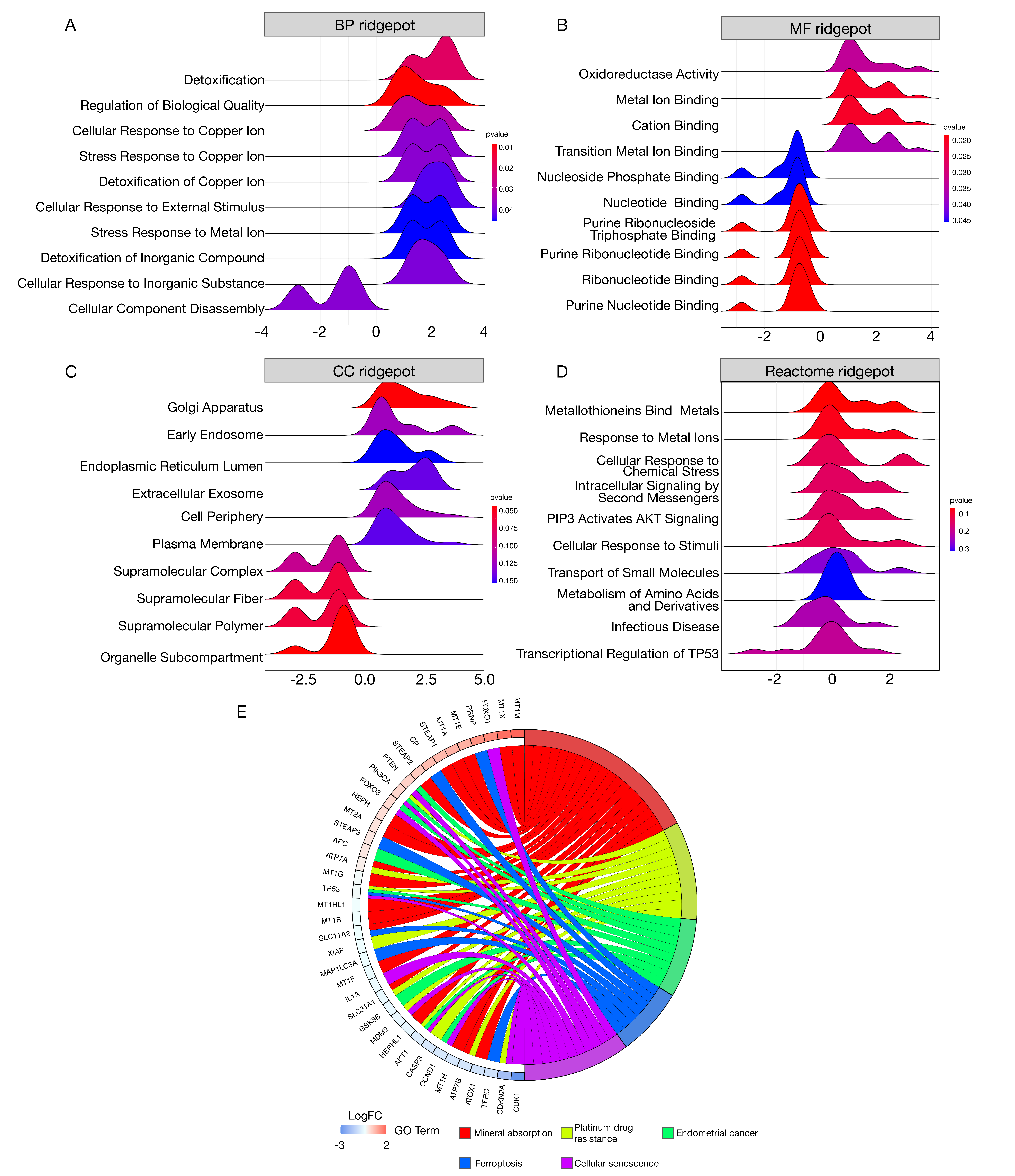

Supplement: Supplementary file 3 — Supplementary Information 3. [file 41598_2024_53560_MOESM3_ESM.tif]

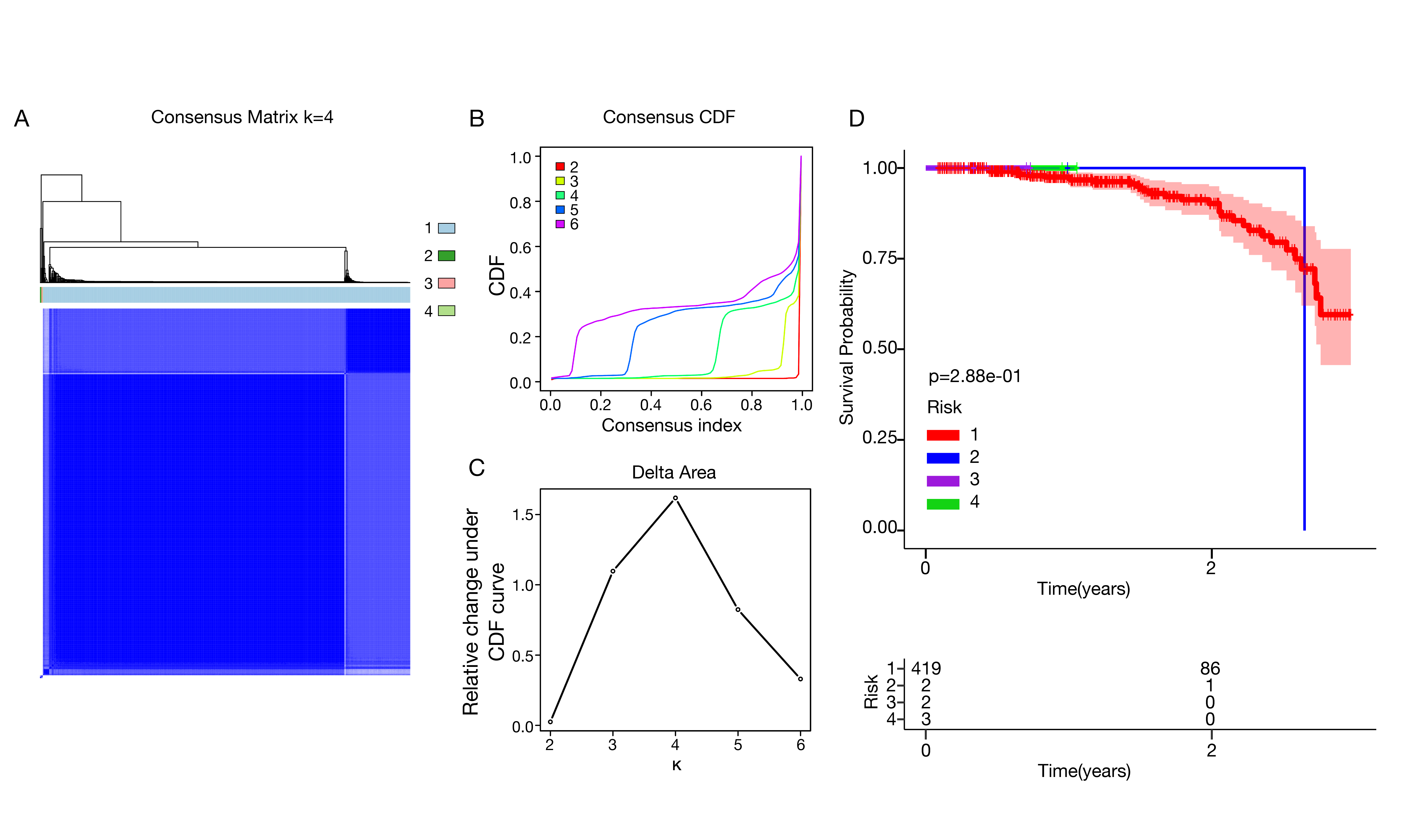

Supplement: Supplementary file 4 — Supplementary Information 4. [file 41598_2024_53560_MOESM4_ESM.tif]

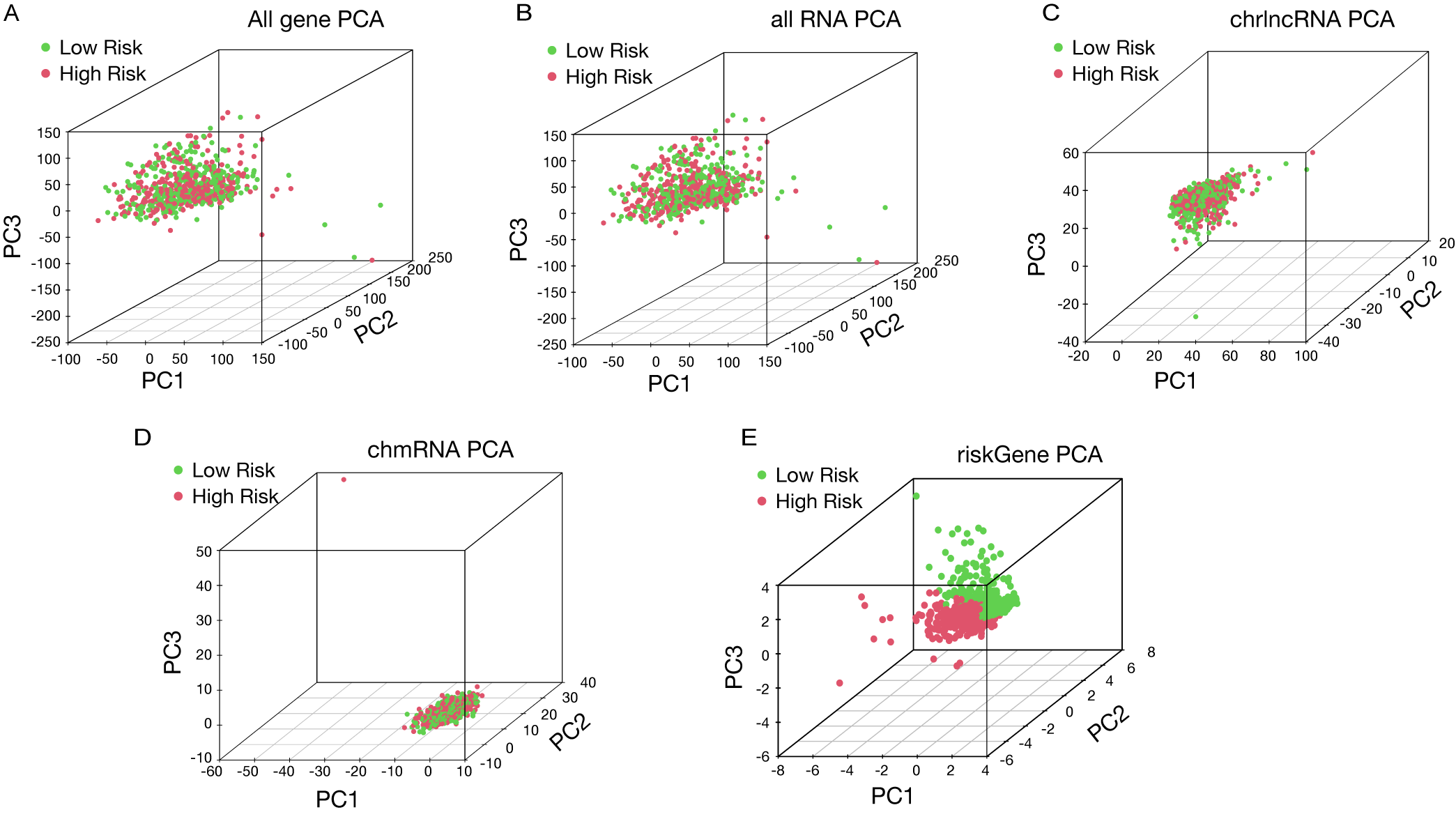

Supplement: Supplementary file 5 — Supplementary Information 5. [file 41598_2024_53560_MOESM5_ESM.tif]

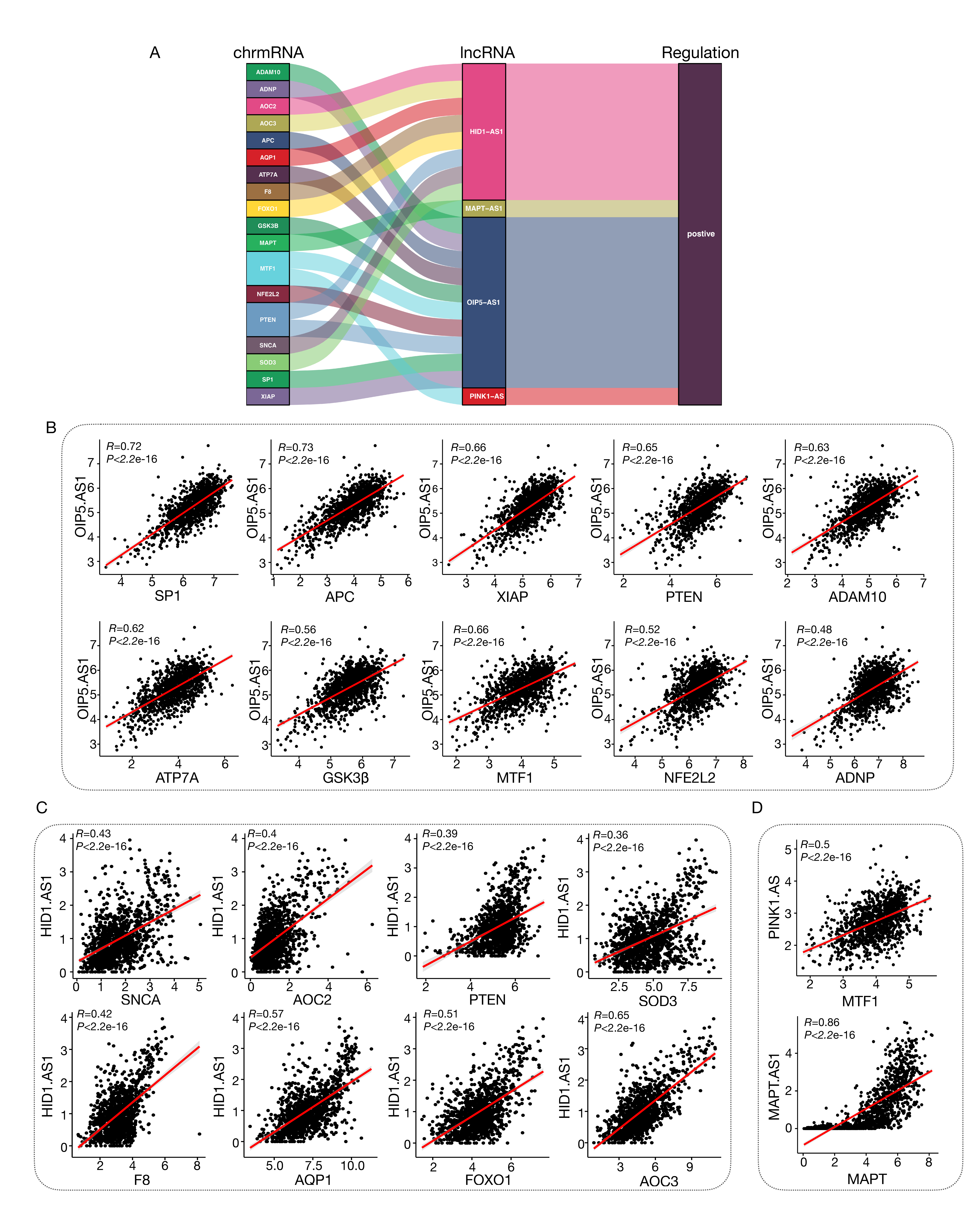

Supplement: Supplementary file 6 — Supplementary Information 6. [file 41598_2024_53560_MOESM6_ESM.tif]

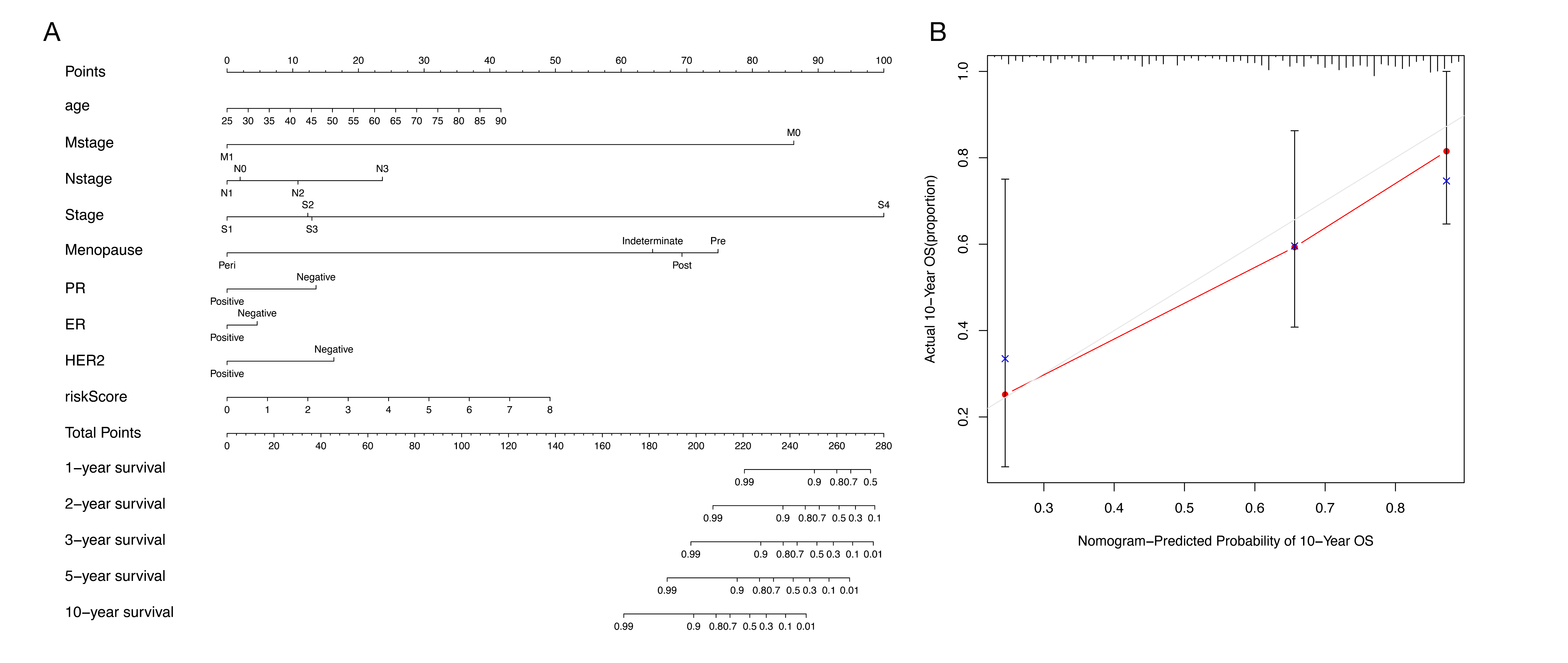

Supplement: Supplementary file 7 — Supplementary Information 7. [file 41598_2024_53560_MOESM7_ESM.tif]

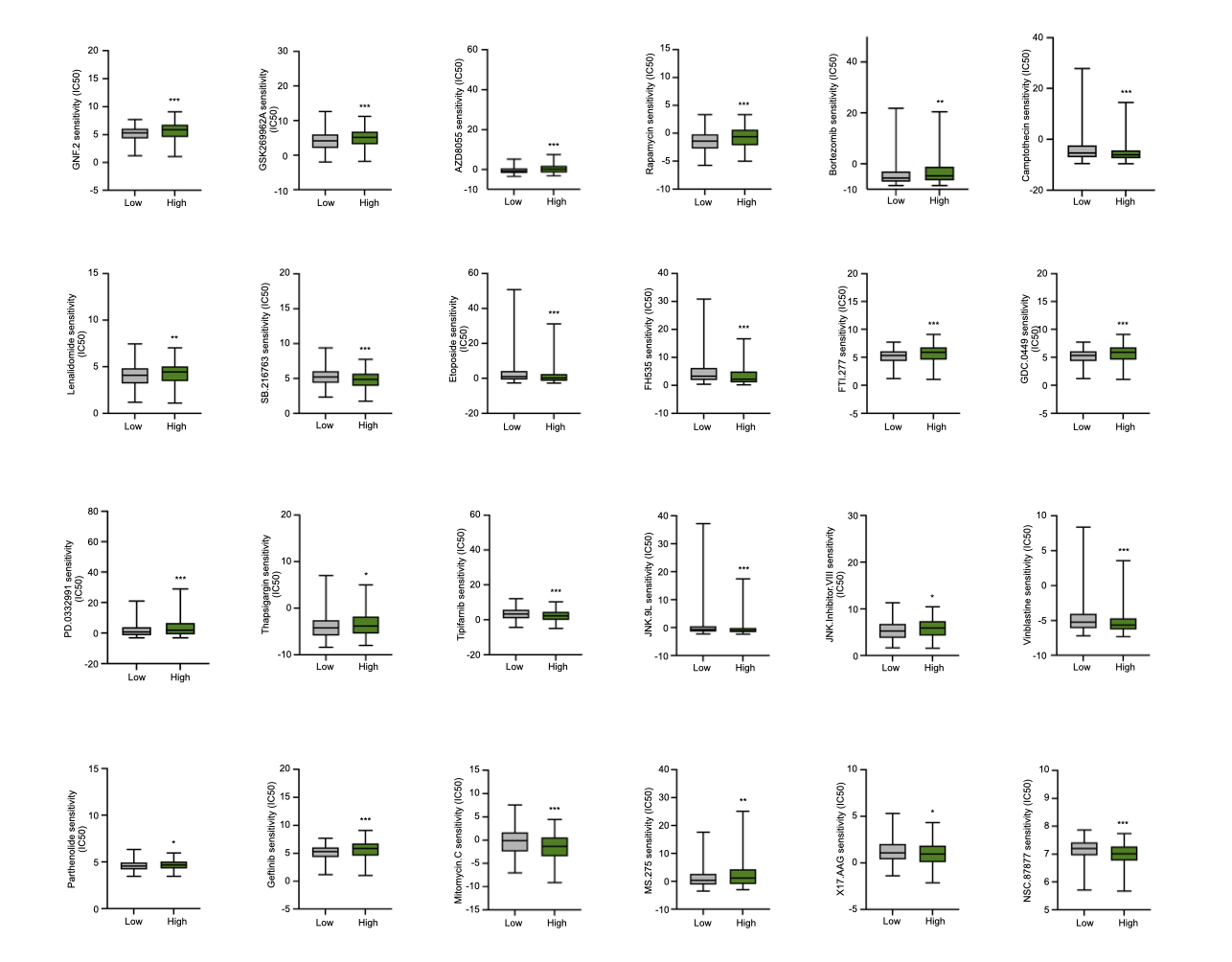

Supplement: Supplementary file 8 — Supplementary Information 8. [file 41598_2024_53560_MOESM8_ESM.tiff]

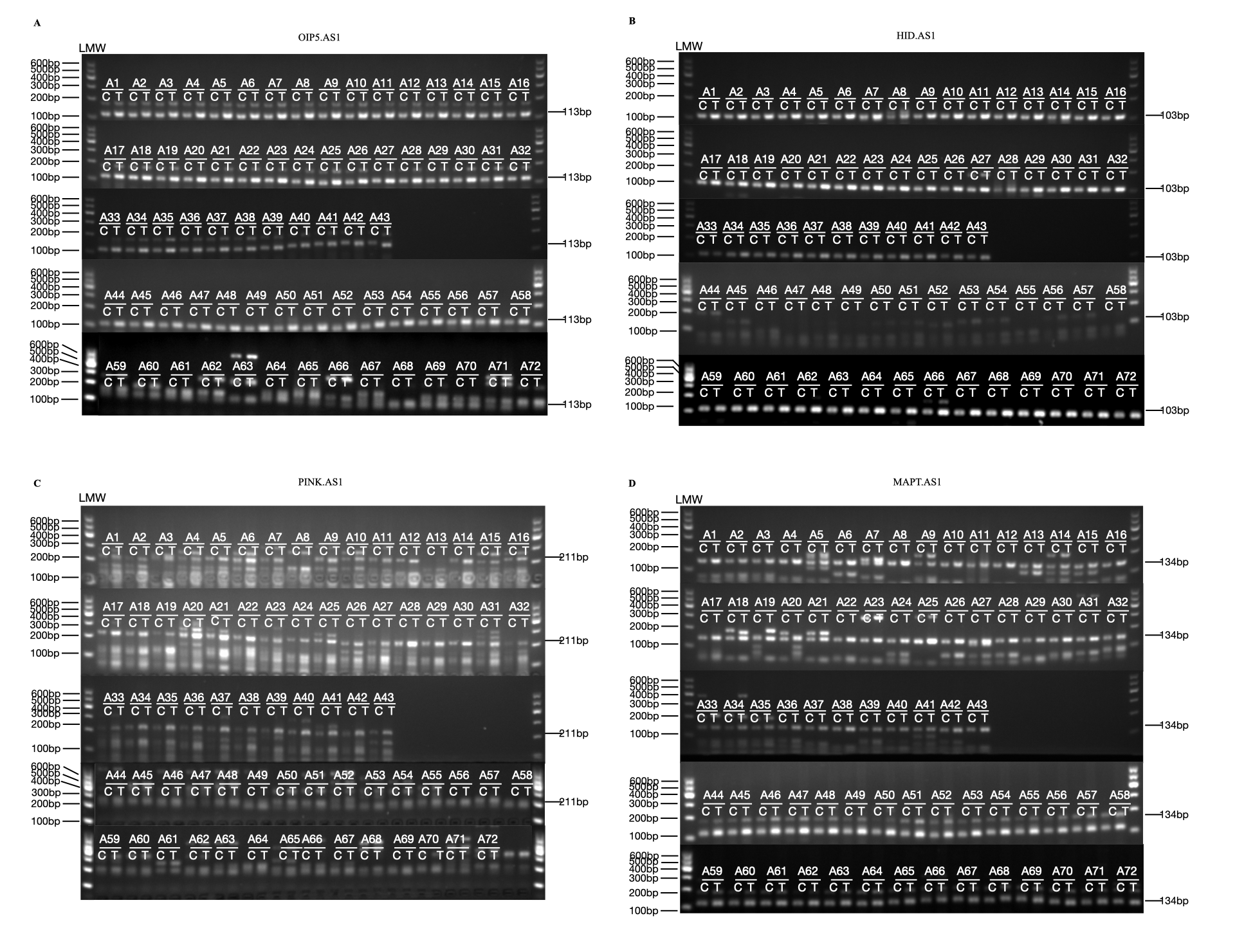

Supplement: Supplementary file 9 — Supplementary Information 9. [file 41598_2024_53560_MOESM9_ESM.tiff]
