# Supplementary material for: Identification and validation of a copper homeostasis-related gene signature for the predicting prognosis of breast cancer patients via integrated bioinformatics analysis
Source: Sci Rep. 2024 Feb 7;14:3141. doi: 10.1038/s41598-024-53560-9 (PMC10850146; doi:10.1038/s41598-024-53560-9)
Supplement: Supplementary file 11 — Supplementary Information 11. [file 41598_2024_53560_MOESM11_ESM.docx]

Supplementary Table 2. Summary of clinical characteristics of 72 patients with primary breast cancer

| Variable | Number of Samples |
| --- | --- |
| Age at diagnosis |  |
| <65/≥65 | 50/22 |
| T stage |  |
| T0/T1/T2/T3/T4 | 1/31/30/7/3 |
| N stage |  |
| N0/N1/N2/N3 | 43/15/7/7 |
| M stage |  |
| M0/M1 | 66/6 |
| AJCC stage |  |
| 0/I/II/III/IV | 1/22/21/27/1 |
| Pathological type |  |
| HR+HER2^-^/ HER2^+^/ Triple negative | 49/14/9 |
| HER2 status |  |
| HER2-0/HER2-low/HER2-positive | 14/43/15 |
| Grading |  |
| 1/2/3 | 7/33/32 |
| Cancer embolus |  |
| No/Yes | 45/27 |
| Perineural invasion |  |
| No/Yes | 62/10 |

AJCC: the American Joint Committee on Cancer; HR: hormone receptor; HER2: human epidermal growth factor receptor 2.
