# Supplementary material for: Identification and validation of a copper homeostasis-related gene signature for the predicting prognosis of breast cancer patients via integrated bioinformatics analysis
Source: Sci Rep. 2024 Feb 7;14:3141. doi: 10.1038/s41598-024-53560-9 (PMC10850146; doi:10.1038/s41598-024-53560-9)
Supplement: Supplementary file 13 — Supplementary Information 13. [file 41598_2024_53560_MOESM13_ESM.docx]

Supplementary Table 4. List of the copper homeostasis and cuprotosis-related genes

| Gene number | Gene name | Description | Relations with GSEA pathway |
| --- | --- | --- | --- |
| 1 | AANAT | Aralkylamine N-Acetyltransferase | GOBP_RESPONSE_TO_COPPER_ION |
| 2 | ABCB6 | ATP Binding Cassette Subfamily B Member 6 (Langereis Blood Group) | GOBP_CELLULAR_COPPER_ION_HOMEOSTASIS  GOBP_COPPER_ION_HOMEOSTASIS |
| 3 | ACR | Acrosin | GOMF_COPPER_ION_BINDING |
| 4 | ADAM10 | ADAM Metallopeptidase Domain 10 | WP_COPPER_HOMEOSTASIS |
| 5 | ADAM17 | ADAM Metallopeptidase Domain 17 | WP_COPPER_HOMEOSTASIS |
| 6 | ADAM9 | ADAM Metallopeptidase Domain 9 | WP_COPPER_HOMEOSTASIS |
| 7 | ADNP | Activity Dependent Neuroprotector Homeobox | GOMF_COPPER_ION_BINDING |
| 8 | AKT1 | AKT Serine/Threonine Kinase 1 | WP_COPPER_HOMEOSTASIS |
| 9 | ALB | Albumin | GOMF_COPPER_ION_BINDING |
| 10 | ANG | Angiogenin | GOMF_COPPER_ION_BINDING |
| 11 | ANKRD9 | Ankyrin Repeat Domain 9 | GOBP_CELLULAR_COPPER_ION_HOMEOSTASIS  GOBP_COPPER_ION_HOMEOSTASIS |
| 12 | AOC1 | Amine Oxidase Copper Containing 1 | GOBP_CELLULAR_RESPONSE_TO_COPPER_ION  GOBP_RESPONSE_TO_COPPER_ION  GOMF_COPPER_ION_BINDING |
| 13 | AOC2 | Amine Oxidase Copper Containing 2 | GOMF_COPPER_ION_BINDING |
| 14 | AOC3 | Amine Oxidase Copper Containing 3 | GOMF_COPPER_ION_BINDING |
| 15 | AP1B1 | Adaptor Related Protein Complex 1 Subunit Beta 1 | HP_ABNORMAL_CIRCULATING_COPPER_CONCENTRATION  HP_DECREASED_CIRCULATING_COPPER_CONCENTRATION |
| 16 | AP1S1 | Adaptor Related Protein Complex 1 Subunit Sigma 1 | HP_ABNORMAL_CIRCULATING_COPPER_CONCENTRATION  HP_DECREASED_CIRCULATING_COPPER_CONCENTRATION |
| 17 | APC | APC Regulator Of WNT Signaling Pathway | WP_COPPER_HOMEOSTASIS |
| 18 | APOA4 | Apolipoprotein A4 | GOMF_COPPER_ION_BINDING |
| 19 | APP | Amyloid Beta Precursor Protein | GOBP_CELLULAR_COPPER_ION_HOMEOSTASIS  GOBP_CELLULAR_RESPONSE_TO_COPPER_ION  GOBP_COPPER_ION_HOMEOSTASIS  GOBP_RESPONSE_TO_COPPER_ION  WP_COPPER_HOMEOSTASIS |
| 20 | AQP1 | Aquaporin 1 (Colton Blood Group) | GOBP_CELLULAR_RESPONSE_TO_COPPER_ION  GOBP_RESPONSE_TO_COPPER_ION |
| 21 | AQP2 | Aquaporin 2 | GOBP_CELLULAR_RESPONSE_TO_COPPER_ION  GOBP_RESPONSE_TO_COPPER_ION |
| 22 | ARF1 | ADP Ribosylation Factor 1 | GOBP_CELLULAR_COPPER_ION_HOMEOSTASIS  GOBP_COPPER_ION_HOMEOSTASIS |
| 23 | ATOX1 | Antioxidant 1 Copper Chaperone | GOBP_CELLULAR_COPPER_ION_HOMEOSTASIS  GOBP_COPPER_ION_HOMEOSTASIS  GOBP_COPPER_ION_TRANSPORT  GOMF_COPPER_ION_BINDING  GOMF_COPPER_CHAPERONE_ACTIVITY  WP_COPPER_HOMEOSTASIS |
| 24 | ATP13A2 | ATPase Cation Transporting 13A2 | GOMF_COPPER_ION_BINDING |
| 25 | ATP5F1D | ATP Synthase F1 Subunit Delta | GOBP_RESPONSE_TO_COPPER_ION |
| 26 | ATP6AP1 | ATPase H+ Transporting Accessory Protein 1 | HP_ABNORMAL_CIRCULATING_COPPER_CONCENTRATION |
| 27 | ATP6V0A2 | ATPase H+ Transporting V0 Subunit A2 | HP_ABNORMAL_CIRCULATING_COPPER_CONCENTRATION |
| 28 | ATP7A | ATPase Copper Transporting Alpha | GOBP_CELLULAR_COPPER_ION_HOMEOSTASIS  GOBP_CELLULAR_RESPONSE_TO_COPPER_ION  GOBP_COPPER_ION_HOMEOSTASIS  GOBP_COPPER_ION_IMPORT  GOBP_COPPER_ION_TRANSMEMBRANE_TRANSPORT  GOBP_COPPER_ION_TRANSPORT  GOBP_DETOXIFICATION_OF_COPPER_ION  GOBP_RESPONSE_TO_COPPER_ION  GOMF_COPPER_ION_BINDING  GOMF_COPPER_CHAPERONE_ACTIVITY  GOMF_COPPER_ION_TRANSMEMBRANE_TRANSPORTER_ACTIVITY  WP_COPPER_HOMEOSTASIS |
| 29 | ATP7B | ATPase Copper Transporting Beta | GOBP_CELLULAR_COPPER_ION_HOMEOSTASIS  GOBP_COPPER_ION_HOMEOSTASIS  GOBP_COPPER_ION_IMPORT  GOBP_COPPER_ION_TRANSMEMBRANE_TRANSPORT  GOBP_COPPER_ION_TRANSPORT  GOBP_RESPONSE_TO_COPPER_ION  GOMF_COPPER_ION_BINDING  GOMF_COPPER_ION_TRANSMEMBRANE_TRANSPORTER_ACTIVITY  WP_COPPER_HOMEOSTASIS |
| 30 | BACE1 | Beta-Secretase 1 | GOBP_CELLULAR_RESPONSE_TO_COPPER_ION  GOBP_RESPONSE_TO_COPPER_ION  WP_COPPER_HOMEOSTASIS |
| 31 | BECN1 | Beclin 1 | GOBP_CELLULAR_RESPONSE_TO_COPPER_ION  GOBP_RESPONSE_TO_COPPER_ION |
| 32 | CASP3 | Caspase 3 | WP_COPPER_HOMEOSTASIS |
| 33 | CCDC22 | Coiled-Coil Domain Containing 22 | GOBP_CELLULAR_COPPER_ION_HOMEOSTASIS  GOBP_COPPER_ION_HOMEOSTASIS |
| 34 | CCND1 | Cyclin D1 | WP_COPPER_HOMEOSTASIS  WP_COPPER_HOMEOSTASIS |
| 35 | CCS | Copper Chaperone For Superoxide Dismutase | GOMF_COPPER_ION_BINDING  WP_COPPER_HOMEOSTASIS |
| 36 | CDK1 | Cyclin Dependent Kinase 1 | GOBP_RESPONSE_TO_COPPER_ION |
| 37 | CDKN2A | Cyclin Dependent Kinase Inhibitor 2A | Peter Tsvetkov et. al. [6] |
| 38 | COA6 | Cytochrome C Oxidase Assembly Factor 6 | GOMF_COPPER_ION_BINDING |
| 39 | COG2 | Component Of Oligomeric Golgi Complex 2 | HP_ABNORMAL_CIRCULATING_COPPER_CONCENTRATION  HP_DECREASED_CIRCULATING_COPPER_CONCENTRATION |
| 40 | COMMD1 | Copper Metabolism Domain Containing 1 | GOBP_COPPER_ION_HOMEOSTASIS  GOMF_COPPER_ION_BINDING  WP_COPPER_HOMEOSTASIS |
| 41 | COX11 | Cytochrome C Oxidase Copper Chaperone COX11 | GOMF_COPPER_ION_BINDING  WP_COPPER_HOMEOSTASIS |
| 42 | COX17 | Cytochrome C Oxidase Copper Chaperone COX17 | GOBP_COPPER_ION_TRANSPORT  GOMF_COPPER_ION_BINDING  GOMF_COPPER_CHAPERONE_ACTIVITY  WP_COPPER_HOMEOSTASIS |
| 43 | COX19 | Cytochrome C Oxidase Assembly Factor COX19 | GOBP_CELLULAR_COPPER_ION_HOMEOSTASIS  GOBP_COPPER_ION_HOMEOSTASIS |
| 44 | CP | Ceruloplasmin | GOBP_COPPER_ION_TRANSPORT  GOMF_COPPER_ION_BINDING  HP_ABNORMAL_CIRCULATING_COPPER_CONCENTRATION  HP_DECREASED_CIRCULATING_COPPER_CONCENTRATION |
| 45 | CUTA | CutA Divalent Cation Tolerance Homolog | GOMF_COPPER_ION_BINDING |
| 46 | CUTC | CutC Copper Transporter | GOBP_COPPER_ION_HOMEOSTASIS  GOBP_COPPER_ION_TRANSPORT  GOMF_COPPER_ION_BINDING |
| 47 | CYP1A1 | Cytochrome P450 Family 1 Subfamily A Member 1 | GOBP_CELLULAR_RESPONSE_TO_COPPER_ION  GOBP_RESPONSE_TO_COPPER_ION |
| 48 | DAXX | Death Domain Associated Protein | GOBP_CELLULAR_RESPONSE_TO_COPPER_ION  GOBP_RESPONSE_TO_COPPER_ION |
| 49 | DBH | Dopamine Beta-Hydroxylase | GOMF_COPPER_ION_BINDING |
| 50 | DCT | Dopachrome Tautomerase | GOMF_COPPER_ION_BINDING |
| 51 | DLAT | Dihydrolipoamide S-Acetyltransferase | Peter Tsvetkov et. al. [6] |
| 52 | DLD | Dihydrolipoamide Dehydrogenase | Peter Tsvetkov et. al. [6] |
| 53 | F5 | Coagulation Factor V | GOMF_COPPER_ION_BINDING |
| 54 | F8 | Coagulation Factor VIII | GOMF_COPPER_ION_BINDING |
| 55 | FDX1 | Ferredoxin 1 | Peter Tsvetkov et. al. [6] |
| 56 | FKBP4 | FKBP Prolyl Isomerase 4 | GOBP_COPPER_ION_TRANSPORT |
| 57 | FOXO1 | Forkhead Box O1 | WP_COPPER_HOMEOSTASIS |
| 58 | FOXO3 | Forkhead Box O3 | WP_COPPER_HOMEOSTASIS |
| 59 | GLS | Glutaminase | Peter Tsvetkov et. al. [6] |
| 60 | GPC1 | Glypican 1 | GOMF_COPPER_ION_BINDING |
| 61 | GSK3B | Glycogen Synthase Kinase 3 Beta | WP_COPPER_HOMEOSTASIS |
| 62 | HAMP | Hepcidin Antimicrobial Peptide | GOMF_COPPER_ION_BINDING |
| 63 | HEPH | Hephaestin | GOBP_COPPER_ION_TRANSPORT |
| 64 | HEPHL1 | Hephaestin Like 1 | GOBP_COPPER_ION_TRANSPORT  GOMF_COPPER_ION_BINDING |
| 65 | HSF1 | Heat Shock Transcription Factor 1 | GOBP_CELLULAR_RESPONSE_TO_COPPER_ION  GOBP_RESPONSE_TO_COPPER_ION |
| 66 | IL1A | Interleukin 1 Alpha | GOBP_RESPONSE_TO_COPPER_ION  GOMF_COPPER_ION_BINDING |
| 67 | JUN | Jun Proto-Oncogene, AP-1 Transcription Factor Subunit | WP_COPPER_HOMEOSTASIS |
| 68 | LACC1 | Laccase Domain Containing 1 | GOMF_COPPER_ION_BINDING |
| 69 | LCAT | Lecithin-Cholesterol Acyltransferase | GOBP_RESPONSE_TO_COPPER_ION |
| 70 | LIAS | Lipoic Acid Synthetase | Peter Tsvetkov et. al. [6] |
| 71 | LIPT1 | Lipoyltransferase 1 | Peter Tsvetkov et. al. [6] |
| 72 | LOX | Lysyl Oxidase | GOMF_COPPER_ION_BINDING |
| 73 | LOXL1 | Lysyl Oxidase Like 1 | GOMF_COPPER_ION_BINDING |
| 74 | LOXL2 | Lysyl Oxidase Like 2 | GOBP_RESPONSE_TO_COPPER_ION  GOMF_COPPER_ION_BINDING |
| 75 | LOXL3 | Lysyl Oxidase Like 3 | GOMF_COPPER_ION_BINDING |
| 76 | LOXL4 | Lysyl Oxidase Like 4 | GOMF_COPPER_ION_BINDING |
| 77 | MAP1LC3A | Microtubule Associated Protein 1 Light Chain 3 Alpha | GOBP_CELLULAR_RESPONSE_TO_COPPER_ION |
| 78 | MAPT | Microtubule Associated Protein Tau | GOBP_RESPONSE_TO_COPPER_ION |
| 79 | MDM2 | MDM2 Proto-Oncogene | WP_COPPER_HOMEOSTASIS |
| 80 | MMGT1 | Membrane Magnesium Transporter 1 | GOBP_COPPER_ION_TRANSPORT |
| 81 | MOXD1 | Monooxygenase DBH Like 1 | GOMF_COPPER_ION_BINDING |
| 82 | MOXD2P | Monooxygenase DBH Like 2, Pseudogene | GOMF_COPPER_ION_BINDING |
| 83 | MT1A | Metallothionein 1A | GOBP_CELLULAR_RESPONSE_TO_COPPER_ION  GOBP_DETOXIFICATION_OF_COPPER_ION  GOBP_RESPONSE_TO_COPPER_ION  WP_COPPER_HOMEOSTASIS |
| 84 | MT1B | Metallothionein 1B | GOBP_CELLULAR_RESPONSE_TO_COPPER_ION  GOBP_DETOXIFICATION_OF_COPPER_ION  GOBP_RESPONSE_TO_COPPER_ION  WP_COPPER_HOMEOSTASIS |
| 85 | MT1DP | Metallothionein 1D, Pseudogene | GOBP_CELLULAR_RESPONSE_TO_COPPER_ION  GOBP_DETOXIFICATION_OF_COPPER_ION  GOBP_RESPONSE_TO_COPPER_ION |
| 86 | MT1E | Metallothionein 1E | GOBP_CELLULAR_RESPONSE_TO_COPPER_ION  GOBP_DETOXIFICATION_OF_COPPER_ION  GOBP_RESPONSE_TO_COPPER_ION  WP_COPPER_HOMEOSTASIS |
| 87 | MT1F | Metallothionein 1F | GOBP_CELLULAR_RESPONSE_TO_COPPER_ION  GOBP_DETOXIFICATION_OF_COPPER_ION  GOBP_RESPONSE_TO_COPPER_ION  WP_COPPER_HOMEOSTASIS |
| 88 | MT1G | Metallothionein 1G | GOBP_CELLULAR_RESPONSE_TO_COPPER_ION  GOBP_DETOXIFICATION_OF_COPPER_ION  GOBP_RESPONSE_TO_COPPER_ION  WP_COPPER_HOMEOSTASIS |
| 89 | MT1H | Metallothionein 1H | GOBP_CELLULAR_RESPONSE_TO_COPPER_ION  GOBP_DETOXIFICATION_OF_COPPER_ION  GOBP_RESPONSE_TO_COPPER_ION  WP_COPPER_HOMEOSTASIS |
| 90 | MT1HL1 | Metallothionein 1H Like 1 | GOBP_CELLULAR_RESPONSE_TO_COPPER_ION  GOBP_DETOXIFICATION_OF_COPPER_ION  GOBP_RESPONSE_TO_COPPER_ION |
| 91 | MT1JP | Metallothionein 1J, Pseudogene | WP_COPPER_HOMEOSTASIS |
| 92 | MT1L | Metallothionein 1L, Pseudogene | WP_COPPER_HOMEOSTASIS |
| 93 | MT1M | Metallothionein 1M | GOBP_CELLULAR_RESPONSE_TO_COPPER_ION  GOBP_DETOXIFICATION_OF_COPPER_ION  GOBP_RESPONSE_TO_COPPER_ION |
| 94 | MT1X | Metallothionein 1X | GOBP_CELLULAR_RESPONSE_TO_COPPER_ION  GOBP_DETOXIFICATION_OF_COPPER_ION  GOBP_RESPONSE_TO_COPPER_ION  WP_COPPER_HOMEOSTASIS |
| 95 | MT2A | Metallothionein 2A | GOBP_CELLULAR_COPPER_ION_HOMEOSTASIS  GOBP_CELLULAR_RESPONSE_TO_COPPER_ION  GOBP_RESPONSE_TO_COPPER_ION  WP_COPPER_HOMEOSTASIS  GOBP_DETOXIFICATION_OF_COPPER_ION  GOBP_COPPER_ION_HOMEOSTASIS |
| 96 | MT3 | Metallothionein 3 | GOBP_CELLULAR_RESPONSE_TO_COPPER_ION  GOBP_DETOXIFICATION_OF_COPPER_ION  9GOBP_RESPONSE_TO_COPPER_ION  GOMF_COPPER_ION_BINDING  WP_COPPER_HOMEOSTASIS |
| 97 | MT4 | Metallothionein 4 | GOBP_CELLULAR_RESPONSE_TO_COPPER_ION  GOBP_DETOXIFICATION_OF_COPPER_ION  GOBP_RESPONSE_TO_COPPER_ION  WP_COPPER_HOMEOSTASIS |
| 98 | MTCO2P12 | MT-CO2 Pseudogene 12 | GOMF_COPPER_ION_BINDING |
| 99 | MTF1 | Metal Regulatory Transcription Factor 1 | WP_COPPER_HOMEOSTASIS  Peter Tsvetkov et. al. [6] |
| 100 | MTF2 | Metal Response Element Binding Transcription Factor 2 | WP_COPPER_HOMEOSTASIS |
| 101 | NFE2L2 | NFE2 Like BZIP Transcription Factor 2 | GOBP_CELLULAR_RESPONSE_TO_COPPER_ION  GOBP_RESPONSE_TO_COPPER_ION |
| 102 | OR5AR1 | Olfactory Receptor Family 5 Subfamily AR Member 1 | GOMF_COPPER_ION_BINDING |
| 103 | P2RX4 | Purinergic Receptor P2X 4 | GOMF_COPPER_ION_BINDING |
| 104 | PAM | Peptidylglycine Alpha-Amidating Monooxygenase | GOBP_RESPONSE_TO_COPPER_ION  GOMF_COPPER_ION_BINDING |
| 105 | PARK7 | Parkinsonism Associated Deglycase | GOBP_DETOXIFICATION_OF_COPPER_ION  GOBP_RESPONSE_TO_COPPER_ION  GOMF_COPPER_ION_BINDING  GOMF_COPPER_CHAPERONE_ACTIVITY |
| 106 | PDHA1 | Pyruvate Dehydrogenase E1 Subunit Alpha 1 | Peter Tsvetkov et. al. [6] |
| 107 | PDHB | Pyruvate Dehydrogenase E1 Subunit Beta | Peter Tsvetkov et. al. [6] |
| 108 | PIK3CA | Phosphatidylinositol-4,5-Bisphosphate 3-Kinase Catalytic Subunit Alpha | WP_COPPER_HOMEOSTASIS |
| 109 | PRND | Prion Like Protein Doppel | GOBP_CELLULAR_COPPER_ION_HOMEOSTASIS  GOBP_COPPER_ION_HOMEOSTASIS  GOMF_COPPER_ION_BINDING |
| 110 | PRNP | Prion Protein | GOBP_CELLULAR_COPPER_ION_HOMEOSTASIS  GOBP_CELLULAR_RESPONSE_TO_COPPER_ION  GOBP_COPPER_ION_HOMEOSTASIS  GOBP_RESPONSE_TO_COPPER_ION  GOMF_COPPER_ION_BINDING  WP_COPPER_HOMEOSTASIS |
| 111 | PTEN | Phosphatase And Tensin Homolog | WP_COPPER_HOMEOSTASIS |
| 112 | RNF7 | Ring Finger Protein 7 | GOMF_COPPER_ION_BINDING |
| 113 | S100A12 | S100 Calcium Binding Protein A12 | GOMF_COPPER_ION_BINDING |
| 114 | S100A13 | S100 Calcium Binding Protein A13 | GOMF_COPPER_ION_BINDING |
| 115 | S100A5 | S100 Calcium Binding Protein A5 | GOBP_RESPONSE_TO_COPPER_ION |
| 116 | SCO1 | Synthesis Of Cytochrome C Oxidase 1 | GOBP_CELLULAR_COPPER_ION_HOMEOSTASIS  GOBP_COPPER_ION_HOMEOSTASIS  GOMF_COPPER_CHAPERONE_ACTIVITY  WP_COPPER_HOMEOSTASIS |
| 117 | SLC11A2 | Solute Carrier Family 11 Member 2 | GOBP_COPPER_ION_TRANSMEMBRANE_TRANSPORT  GOBP_COPPER_ION_TRANSPORT  GOMF_COPPER_ION_TRANSMEMBRANE_TRANSPORTER_ACTIVITY  WP_COPPER_HOMEOSTASIS |
| 118 | SLC31A1 | Solute Carrier Family 31 Member 1 | GOBP_CELLULAR_COPPER_ION_HOMEOSTASIS  GOBP_COPPER_ION_HOMEOSTASIS  GOBP_COPPER_ION_IMPORT  GOBP_COPPER_ION_TRANSMEMBRANE_TRANSPORT  GOBP_COPPER_ION_TRANSPORT  GOMF_COPPER_ION_TRANSMEMBRANE_TRANSPORTER_ACTIVITY  WP_COPPER_HOMEOSTASIS |
| 119 | SLC31A2 | Solute Carrier Family 31 Member 2 | GOBP_CELLULAR_COPPER_ION_HOMEOSTASIS  GOBP_COPPER_ION_HOMEOSTASIS  GOBP_COPPER_ION_TRANSMEMBRANE_TRANSPORT  GOBP_COPPER_ION_TRANSPORT  GOMF_COPPER_ION_TRANSMEMBRANE_TRANSPORTER_ACTIVITY  WP_COPPER_HOMEOSTASIS |
| 120 | SNAI3 | Snail Family Transcriptional Repressor 3 | GOBP_RESPONSE_TO_COPPER_ION |
| 121 | SNCA | Synuclein Alpha | GOBP_CELLULAR_RESPONSE_TO_COPPER_ION  GOBP_RESPONSE_TO_COPPER_ION |
| 122 | SNCB | Synuclein Beta | GOBP_RESPONSE_TO_COPPER_ION |
| 123 | SNCG | Synuclein Gamma | GOBP_RESPONSE_TO_COPPER_ION |
| 124 | SOD1 | Superoxide Dismutase 1 | GOBP_RESPONSE_TO_COPPER_ION  WP_COPPER_HOMEOSTASIS |
| 125 | SOD3 | Superoxide Dismutase 3 | GOBP_RESPONSE_TO_COPPER_ION  WP_COPPER_HOMEOSTASIS |
| 126 | SORD | Sorbitol Dehydrogenase | GOBP_RESPONSE_TO_COPPER_ION |
| 127 | SP1 | Sp1 Transcription Factor | WP_COPPER_HOMEOSTASIS |
| 128 | SPATA5 | Spermatogenesis Associated 5 | HP_ABNORMAL_CIRCULATING_COPPER_CONCENTRATION |
| 129 | STEAP1 | STEAP Family Member 1 | WP_COPPER_HOMEOSTASIS |
| 130 | STEAP2 | STEAP2 Metalloreductase | GOBP_COPPER_ION_IMPORT  GOBP_COPPER_ION_TRANSMEMBRANE_TRANSPORT  GOBP_COPPER_ION_TRANSPORT  WP_COPPER_HOMEOSTASIS |
| 131 | STEAP3 | STEAP3 Metalloreductase | GOBP_COPPER_ION_IMPORT  GOBP_COPPER_ION_TRANSPORT  WP_COPPER_HOMEOSTASIS |
| 132 | STEAP4 | STEAP4 Metalloreductase | GOBP_COPPER_ION_IMPORT  GOBP_COPPER_ION_TRANSPORT  WP_COPPER_HOMEOSTASIS |
| 133 | SUMF1 | Sulfatase Modifying Factor 1 | GOBP_RESPONSE_TO_COPPER_ION |
| 134 | TFRC | Transferrin Receptor | GOBP_RESPONSE_TO_COPPER_ION |
| 135 | TMPRSS6 | Transmembrane Serine Protease 6 | HP_ABNORMAL_CIRCULATING_COPPER_CONCENTRATION  HP_DECREASED_CIRCULATING_COPPER_CONCENTRATION |
| 136 | TP53 | Tumor Protein P53 | WP_COPPER_HOMEOSTASIS |
| 137 | TYR | Tyrosinase | GOBP_RESPONSE_TO_COPPER_ION |
| 138 | XAF1 | XIAP Associated Factor 1 | WP_COPPER_HOMEOSTASIS |
| 139 | XIAP | X-Linked Inhibitor Of Apoptosis | GOBP_COPPER_ION_HOMEOSTASIS  WP_COPPER_HOMEOSTASIS |
